# Grading and assessment of clinical predictive tools for paediatric head injury: a new evidence-based approach

**DOI:** 10.1186/s12873-019-0249-y

**Published:** 2019-06-14

**Authors:** Mohamed Khalifa, Blanca Gallego

**Affiliations:** 1 Australian Institute of Health Innovation, Faculty of Medicine and Health Sciences, Macquarie University, 75 Talavera Road, North Ryde, Sydney, NSW 2113 Australia; 2 Centre for Big Data Research in Health, Faculty of Medicine, University of New South Wales, Lowy Cancer Research Centre, Level 4, Cnr High &, Botany St, Kensington, Sydney, NSW 2052 Australia

**Keywords:** Paediatric head injury, Clinical prediction, Clinical decision support, Grading and assessment, Evidence-based, Emergency medicine

## Abstract

**Background:**

Many clinical predictive tools have been developed to diagnose traumatic brain injury among children and guide the use of computed tomography in the emergency department. It is not always feasible to compare tools due to the diversity of their development methodologies, clinical variables, target populations, and predictive performances. The objectives of this study are to grade and assess paediatric head injury predictive tools, using a new evidence-based approach, and to provide emergency clinicians with standardised objective information on predictive tools to support their search for and selection of effective tools.

**Methods:**

Paediatric head injury predictive tools were identified through a focused review of literature. Based on the critical appraisal of published evidence about predictive performance, usability, potential effect, and post-implementation impact, tools were evaluated using a new framework for grading and assessment of predictive tools (GRASP). A comprehensive analysis was conducted to explain why certain tools were more successful.

**Results:**

Fourteen tools were identified and evaluated. The highest-grade tool is PECARN; the only tool evaluated in post-implementation impact studies. PECARN and CHALICE were evaluated for their potential effect on healthcare, while the remaining 12 tools were only evaluated for predictive performance. Three tools; CATCH, NEXUS II, and Palchak, were externally validated. Three tools; Haydel, Atabaki, and Buchanich, were only internally validated. The remaining six tools; Da Dalt, Greenes, Klemetti, Quayle, Dietrich, and Güzel did not show sufficient internal validity for use in clinical practice.

**Conclusions:**

The GRASP framework provides clinicians with a high-level, evidence-based, comprehensive, yet simple and feasible approach to grade, compare, and select effective predictive tools. Comparing the three main tools which were assigned the highest grades; PECARN, CHALICE and CATCH, to the remaining 11, we find that the quality of tools’ development studies, the experience and credibility of their authors, and the support by well-funded research programs were correlated with the tools’ evidence-based assigned grades, and were more influential, than the sole high predictive performance, on the wide acceptance and successful implementation of the tools. Tools’ simplicity and feasibility, in terms of resources needed, technical requirements, and training, are also crucial factors for their success.

**Electronic supplementary material:**

The online version of this article (10.1186/s12873-019-0249-y) contains supplementary material, which is available to authorized users.

## Background

Clinical decision support (CDS) systems proved to enhance evidence-based clinical practice and improve healthcare cost-effectiveness [1–6]. Based on Shortliffe’s three levels classification, clinical predictive tools, here referred to simply as predictive tools, belong to the highest CDS level; providing patient-specific recommendations based on clinical scenarios, which usually follow clinical rules and algorithms, cost benefit analysis, or clinical pathways [7, 8]. These research-based applications quantify the contributions of relevant patient characteristics to derive the likelihood of diseases, predict their courses and possible outcomes, or support the decision making on their management [9–11]. Among the healthcare areas that are increasingly utilising predictive tools is the emergency department (ED) [11, 12]. Some of these tools have been demonstrated to support EDs to overcome many of the encountered challenges, such as overcrowding of patients, lack of resources, variable acuity and diversity of clinical conditions [13, 14]. They also have the potential to help clinicians to improve effectiveness through achieving better clinical outcomes, improve efficiency by reducing costs, and improve patient safety by minimising complications and unintended consequences [15–17].

Traumatic brain injury (TBI) is one of the most commonly presenting emergency conditions and is the leading cause of death and disability among trauma patients [18, 19]. In 2017, the centers for disease control and prevention (CDC) reported that the annual TBI related ED visits were estimated at 2.5 million incidents in the United States (US) [20]. Approximately, one third of these incidents occurred among children aged 0 to 14 years [21]. Many predictive tools have been developed, over the last 25 years, to support the diagnosis of TBI among children and guide the use of computed tomography (CT) in the ED [22, 23]. Through predicting TBI and identifying children who are at low risk of clinically important incidents, these tools are designed to decrease CT scan over-utilisation, to save time and money, and to minimise the exposure of children to the harmful ionising radiation, without compromising their safety or missing clinically significant events [24–28].

When selecting a predictive tool, for implementation at their clinical practice or for recommendation in clinical practice guidelines, clinicians involved in the decision making are challenged with an overwhelming and ever-growing number of tools. Many of these tools have never been implemented or assessed for comparative effectiveness or post-implementation impact [29–31]. Currently, clinicians rely on their previous experience, subjective evaluation or recent exposure to predictive tools in making selection decisions. Objective methods and evidence based approaches are rarely used in such decisions [32, 33]. Some clinicians, especially those developing clinical guidelines, search the literature for the best available published evidence. Commonly they look for studies that describe the development, implementation or evaluation of predictive tools. More specifically, some clinicians look for systematic reviews on predictive tools, comparing their development processes or predictive performances. However, there are no available methods to objectively and comprehensively summarise and interpret such evidence [34, 35].

While there are many predictive tools that have been developed, to help clinicians rule out TBI among children at the ED, only a few were considered for use in clinical practice [22–24]. Therefore, we need to understand what makes certain tools more widely accepted and successfully implemented than the others. This will help national and institutional guideline developer clinicians to make better decisions in selecting and incorporating effective predictive tools in their clinical guidelines to help other clinicians through the decision-making process. Furthermore, this will also help expert clinicians develop better predictive tools for the clinical practice in the future. In addition to the predictive performance measures, such as the sensitivities and specificities of predictive tools, many other quantitative and qualitative measures can be considered for the analysis. The country and year of tools’ development could have an influence on the tools’ acceptance and success. In addition, the number of citations and studies that report the tools’ validation, evaluation or implementation could indicate some sort of attention and acceptance. Furthermore, the quality of the tools’ development studies, and the efforts invested in their development, reflected in the sample size of patients or records used in the development and the number of authors and their experiences, could support tools’ wide acceptance and successful implementation.

The primary objective of this study is to grade and assess paediatric head injury predictive tools using a new evidence-based framework for grading and assessment of predictive tools (The GRASP Framework). The secondary objective is to provide emergency clinicians with standardised objective information on clinical predictive tools to support their search for and selection of effective tools.

## Methods

Our study is composed of three parts. The first includes identifying paediatric head injury predictive tools, proposed in the literature, and their related published evidence. The second part includes grading these predictive tools using our new evidence-based approach and eligible published evidence. The third part includes conducting a comprehensive and objective analysis to answer the research question.

### Identifying predictive tools

We conducted a focused review of the literature on paediatric head injury predictive tools. The concepts used in the literature search included “paediatrics”, “head”, “injury”, “clinical prediction”, “tools”, “rules”, “models”, “development”, “validation”, “implementation”, and “evaluation”. The search was conducted for studies published in English language, with no specific time frame, using MEDLINE, EMBASE, CINAHL, and Google Scholar. The default time range of each database was used, including available publications since 1879, 1950, 1947, and 1937 respectively and up to January 2019. The search followed five steps. 1) Systematic reviews on paediatric head injury predictive tools were identified and retrieved. 2) Examining the systematic reviews; the primary studies, describing the development of the tools, were then identified and retrieved. 3) All secondary studies that cited the primary studies or that referred to the tools’ names or to any of their authors, anywhere in the text, were retrieved. 4) All tertiary studies that cited the secondary studies or that were used as references by the secondary studies were retrieved. 5) Secondary and tertiary studies were examined to exclude non-relevant studies or those not reporting the validation, implementation or evaluation of the tools. Additional file 1: Figure S2 shows the process of searching the literature for the paediatric head injury predictive tools and their related published evidence.

### Grading predictive tools

Each paediatric head injury predictive tool was evaluated using our newly developed framework for grading and assessment of predictive tools (abbreviated as GRASP) [36]. Eligible studies were examined in detail for the reported evaluations of the predictive tools. Based on the critical appraisal of the published evidence on predictive tools, the GRASP framework uses three dimensions to grade predictive tools: 1) Phase of Evaluation, 2) Level of Evidence and 3) Direction of Evidence.

#### Phase of evaluation

Assigns A, B, or C based on the highest phase of evaluation. If a tool’s predictive performance, as reported in the literature, has been tested for validity, it is assigned phase C. If a tool’s usability and/or potential effect have been tested, it is assigned phase B. Finally, if a tool has been implemented in the clinical practice, and there is published evidence evaluating its post-implementation impact, it is assigned phase A.

#### Level of evidence

A numerical score, within each phase, is assigned based on the level of evidence associated with each tool. A tool is assigned grade C1 if it has been tested for external validity multiple times, grade C2 if it has been tested for external validity only once, and grade C3 if it has been tested only for internal validity. Grade C0 means that the tool did not show sufficient internal validity to be used in the clinical practice. Grade B1 is assigned to a predictive tool that has been evaluated, during the planning for implementation, for both of its potential effect, on clinical effectiveness, patient safety or healthcare efficiency, and for its usability. Grade B2 is assigned to a predictive tool that has been evaluated only for its potential effect, while if it has been studied only for its usability, it is assigned grade B3. Finally, if a predictive tool had been implemented then evaluated for its post-implementation impact, on clinical effectiveness, patient safety or healthcare efficiency, then it is assigned grade A1 if there is at least one experimental study of good quality evaluating its post-implementation impact, grade A2 if there are observational studies evaluating its impact, and grade A3 if the post-implementation impact has been evaluated only through subjective studies, such as expert panel reports.

#### Direction of evidence

For each phase and level of evidence, a direction of evidence is assigned based on the collective conclusions reported in the studies. The evidence is considered positive if all studies about a predictive tool reported positive conclusions and negative if all studies reported negative or equivocal conclusions. The evidence is considered mixed if some studies reported positive and some reported either negative or equivocal conclusions. To decide an overall direction of evidence, a protocol is used to sort the mixed evidence into 1) Mixed evidence that supports an overall positive conclusion or 2) Mixed evidence that supports an overall negative conclusion. This protocol is based on two main criteria; 1) Degree of matching between the evaluation study conditions and the original tool specifications, and 2) Quality of the evaluation study. Studies evaluating predictive tools in closely matching conditions to the tool specifications and providing high quality evidence are considered first; taking into account their conclusions in deciding the overall direction of evidence.

**Fig. 1:**
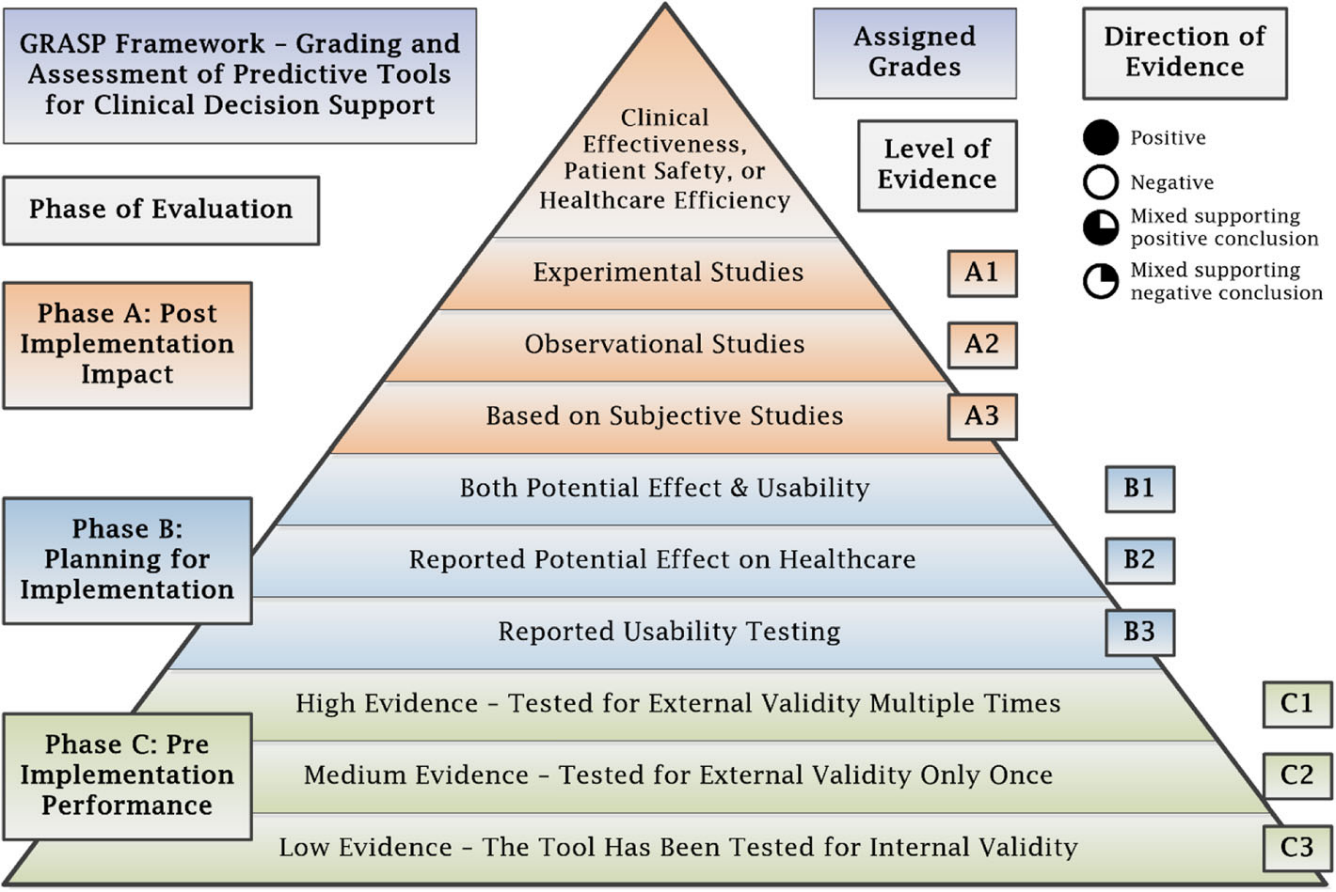
The GRASP Framework Concept [36]

The final grade assigned to a tool is based on the highest phase of evaluation, supported by the highest level of positive evidence, or mixed evidence that supports a positive conclusion. The GRASP framework concept is shown in Fig. 1 and the GRASP framework detailed report is presented in Additional file 1: Table S3.

## Results

### Identifying predictive tools

We identified five systematic reviews [22–24, 27, 28] and two literature reviews [37, 38] discussing paediatric head injury predictive tools. Through these seven reviews, we identified 16 studies describing the development and internal validation of 14 distinct predictive tools [39–54]. After development and internal validation, the PECARN rule (Paediatric Emergency Care Applied Research Network) [49] was evaluated in 23 studies [55–77]. The CHALICE rule (Children’s Head injury ALgorithm for the prediction of Important Clinical Events) [43] was evaluated in 13 studies [24, 48, 58–62, 66, 69, 72, 77–80]. The CATCH rule (Canadian Assessment of Tomography for Childhood Head injury) [51] was evaluated in 11 studies [48, 58–61, 63, 66, 72, 81–83]. The NEXUS II rule (National Emergency X-Radiography Utilization Study) [50, 54] was evaluated in four studies [48, 84–86]. Palchak rule [52] was evaluated in two studies [48, 87]. On the other hand, none of the remaining nine rules; Haydel [47], Atabaki [39], Buchanich [40], Da Dalt [41], Greenes [44, 45], Klemetti [48], Quayle [53], Dietrich [42], or Güzel [46] were evaluated in published studies after their initial development.

### Grading predictive tools

Using the GRASP framework and eligible evidence, we assigned grades to the 14 paediatric head injury predictive tools. The **PECARN** rule was developed by Dr. Nathan Kuppermann in the US in 2009 and was tested successfully for internal validity [49]. The rule was tested multiple times for external validity and proved externally valid in all the reported studies [56, 58–61, 63, 66, 67, 70–74, 76, 77]. This qualifies the PECARN rule for grade C1. Four economic analysis studies discussed the positive potential effects of using the PECARN rule on lowering healthcare costs, decreasing the frequency of using CT scans and minimising the exposure of children to harmful ionising radiation [62, 68, 69, 75]. This qualifies the PECARN rule for grade B2. Three observational post-implementation impact studies were conducted. One study concluded that the PECARN intermediate-risk predictors did not play a major role in the physicians’ decision to perform a CT scan [65]. However, the other two studies concluded that implementing and using the PECARN rule was associated with a statistically significant decrease in CT utilisation without safety or effectiveness issues [57, 64]. Using the protocol, the mixed evidence here supports positive conclusion on the post-implementation impact of the PECARN rule. Accordingly, the final grade assigned to the PECARN rule is A2.

The **CHALICE** rule was developed by Dr. Joel Dunning in the United Kingdom in 2006 and was tested successfully for internal validity [43]. The rule was tested multiple times for external validity and proved externally valid in all the reported studies [48, 58–61, 66, 72, 77]. This qualifies the CHALICE rule for grade C1. Six cost-effectiveness studies discussed the potential effects of implementing the rule; whether it would increase or decrease the number and costs of CT scans and its potential effect on the exposure of children to radiation. Two of the six studies in 2010 reported that the implementation of CHALICE rule would increase the number of CT scans performed and increase the exposure of children to the harmful ionising radiation [79, 80]. However, four subsequent studies in 2011, 2013, 2015 and 2016 reported that implementing the CHALICE rule would be a cost-effective strategy to safely reduce unnecessary head CT scans [24, 62, 69, 78]. Using the protocol, the mixed evidence here supports positive conclusion on the cost-effectiveness and potential effects of implementing the CHALICE rule. The rule was not evaluated for usability or post-implementation impact. Accordingly, the final grade assigned to the CHALICE rule is B2.

The **CATCH** rule was developed by Dr. Martin Osmond in the US in 2010 and was tested successfully for internal validity [51]. The rule was tested multiple times for external validity and proved externally valid in all the reported studies [48, 58–61, 63, 66, 72, 81]. The rule was not evaluated for usability, potential effect or post-implementation impact. Accordingly, the final grade assigned to the CATCH rule is C1.

The **NEXUS II** rule was developed by Dr. William Mower in the US in 2005, primarily for the diagnosis of adult head injury [88, 89]. Later on, the rule was validated for paediatrics by Dr. Jennifer Oman in the US in 2006 [50]. The tool was then tested multiple times for external validity. One study failed to properly evaluate the rule after using a modified version, which did not show external validity [54]. Two studies proved the rule was externally valid for children less than 14 and 16 years [48, 85] and one study proved the rule was externally valid for children over 10 years [86]. Using the protocol, the mixed evidence here supports positive conclusion on external validity. The rule was not evaluated for usability, potential effect or post-implementation impact. Accordingly, the final grade assigned to the NEXUS II rule is C1.

**Palchak** rule was developed by Dr. Michael Palchak and Dr. Nathan Kuppermann in the US in 2003 and was tested successfully for internal validity [52]. A study by the same authors in 2009 included validation of the rule in comparison to clinicians’ judgement using the same dataset that was used for the rule development, so this is still considered an internal validation [87]. One external validation study reported the predictive performance of Palchak rule was acceptable [48]. The rule was not evaluated for usability, potential effect or post-implementation impact. Accordingly, the final grade assigned to Palchak rule is C2.

**Haydel** rule was developed by Dr. Micelle Haydel in the US in 2003 [47], **Atabaki** rule was developed by Dr. Shireen Atabaki in the US in 2008 [39], and **Buchanich** rule was developed by Dr. Jeanine Buchanich in the US in 2007 [40]. The three rules were tested successfully for internal validity. However, they were not tested for external validity; neither were they evaluated for usability, potential effect or post-implementation impact. Accordingly, the final grade assigned to these three rules is C3.

**Da Dalt** rule was developed by Dr. Liviana Da Dalt in Italy in 2006 [41], **Greenes** rule was developed by Dr. David Greenes in the US in 2001 [44, 45], and **Klemetti** rule was developed Dr. Sanna Klemetti in Finland in 2009 [48]. The studies conducted by these three researchers followed correct development methods for their proposed tools. However, the internal validation processes of the tools were not clearly reported. Accordingly, the final grade assigned to these three rules is C0.

**Dr. Kimberly Quayle** in the US in 1997 [53], **Dr. Ann Dietrich** in the US in 1993 [42], and **Dr. Ahmet Güzel** in Turkey in 2009 [46], each tried to develop a clinical prediction rule to identify children at low risk for traumatic brain injury after head trauma. Their studies discussed clinical risk factors, symptoms and signs that could reliably predict abnormalities in cranial computed tomography (CT) scans. Even though each used a different mix of common clinical variables, none of the three studies could demonstrate sufficient correlations between clinical variables, symptoms and signs of significant TBI and the later findings on CT.

Therefore, they could not produce predictive rules with sufficient internal validity. Accordingly, the final grade assigned to these three rules is C0. A summary of the results of grading the 14 paediatric head injury predictive tools, using the GRASP framework, is presented in Table 1. The GRASP framework detailed reports, of each of the 14 paediatric head injury predictive tools, are presented in Additional file 1: Tables S4 to S17.

**Table 1:**
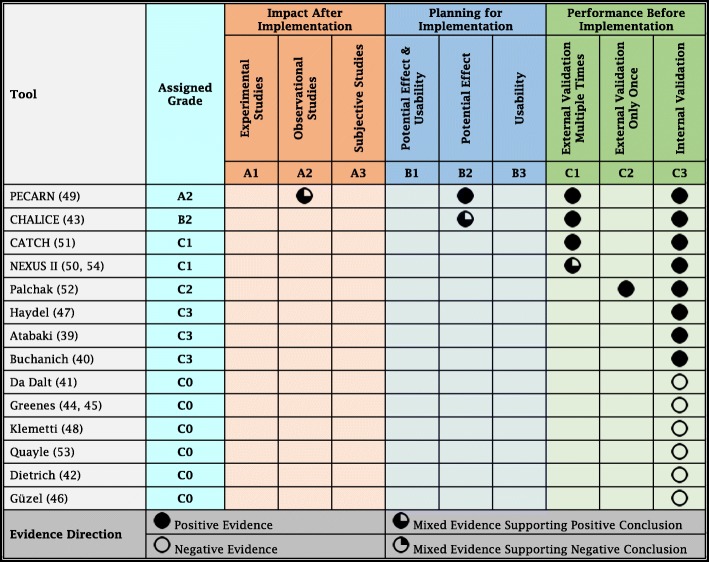
Summary of Grading Paediatrics Head Injury Predictive Tools

### Findings of the tools’ analysis

The PECARN rule was the only tool evaluated in post-implementation impact studies. The PECARN and the CHALICE rules were evaluated for potential effect on healthcare, while the remaining 12 tools were only evaluated for predictive performance. Three of these 12 tools were externally validated; CATCH, NEXUS II, and Palchak rules, three were only internally validated; Haydel, Atabaki, and Buchanich rules, and the remaining six tools; Da Dalt, Greenes, Klemetti, Quayle, Dietrich, and Güzel rules did not show sufficient internal validity to be used in clinical practice.

Using statistical analysis, we explored possible correlations between different criteria of predictive tools and their evidence-based assigned grades. There is no correlation between the country of the tools’ development and their assigned grades. For example, the 10 tools developed in the US include some of the highest and some of the lowest grades, so the country of a tool’s development is not related to the grade of the tool. There is a weak correlation between the year of the tools’ development and their assigned grades. The tools developed more recently could be higher in grade. There is a strong correlation between the number of citations of the tools, in the literature, and their assigned grades. The tools with higher citations are expected to be higher in grade. There is a very strong correlation between the number of studies discussing the tools and their assigned grades. The tools discussed and reported in more studies are higher in grade.

To provide clinicians with a few more objective measures to compare the tools, in addition to the citations and the published studies, we developed three derived values; the citation index, the publication index, and the literature index. The PECARN, the CHALICE and the CATCH rules were cited in the literature 885, 309, and 319 times respectively. To make these figures comparable, we calculated the citation index as the average annual citations for each tool, by dividing the total citations of each tool by its age in years. Similarly, the publication index is the average annual studies discussing each tool. We also calculated a literature index; by multiplying the total number of citations by the total number of studies, on each tool, divided by 1000, for simplification. This figure reflects the post-implementation impact of each tool in the literature. Like the citations and publications, the three indices of the tools are strongly correlated with their assigned grades.

Looking at more detailed objective measures, reported in the development studies of the 14 paediatric head injury predictive tools; we find very interesting results. The predictive tools were developed using two main methodologies. Recursive partitioning was used to develop the PECARN, CHALICE, CATCH, NEXUS II, Palchak, Haydel, Atabaki, and Buchanich rules. Multivariate logistic regression analysis was used to develop Greenes, Da Dalt, Klemetti, Quayle, Dietrich and Güzel rules. In addition, many clinical variables were used in the development of the tools, such as altered mental status, amnesia, focal neurological signs, occurrence of seizure after injury, presence of skull fractures, loss of consciousness, history of headache and/or vomiting. The mix of clinical variables used, to build the tools’ predictive models and their outcome scores, were similar but not the same for any of the tools. Moreover, the tools development studies used different paediatric populations and sample sizes. Consequently, the predictive performances of the tools, such as their sensitivities and specificities, were variable. Most of the tools showed high sensitivities, with the majority ranging from 90 to 100%, while their specificities were very different; ranging from 15 to 87%.

There is no correlation between the tools’ development methodologies and their predictive performances. However, most of the tools developed using recursive partitioning showed relatively higher sensitivities but not necessarily better specificities. In addition, there is no correlation between the tools’ development methodologies and their assigned grades. However, the six tools that used multivariate logistic regression analysis were all assigned grade C0; reporting no internal validity, while the other eight tools that used recursive partitioning showed higher variable grades. Furthermore, there is no correlation between the predictive performances of the tools and their assigned grades. For example, Da Dalt rule is assigned grade C0.

However, it has the highest sensitivity of 100% and the highest specificity of 87% among all the tools. This could be explained by the fact that Da Dalt rule was not internally validated, which makes it unqualified for external validation or implementation. While the CHALICE rule, which is assigned grade B2, has a sensitivity of 98% and a specificity of 86%, we find that the PECARN rule, which is the highest tool, assigned grade A2, has a similar sensitivity of 97% but lower specificity of 59%.

On the other hand, we find that there is a strong correlation between the size of the patient samples used in the development and internal validation studies of the tools and their assigned grades. The three main tools had the largest numbers of patients contributing to their development studies; 42,412 patients were enrolled and analysed to develop the PECARN rule, 22,772 to develop the CHALICE, and 3866 to develop the CATCH rule. The remaining 11 tools were developed using a relatively smaller number of patient samples, ranging from 3000 to only a hundred patients. In addition, there is a strong correlation between the number of researchers developing tools and their assigned grades. Two of the main three tools were developed by a large number of researchers; the PECARN rule was developed by 32 researchers and the CATCH rule was developed by 14 researchers. The remaining tools were developed by a relatively fewer number of researchers; ranging from 10 for the Palchak rule to only one researcher for the Buchanich rule.

Moreover, there is a correlation between the impact factor of the journal that published the development studies of the tools and their assigned grades. The PECARN rule, for example, was published in the Lancet, which is a highly ranked journal with an impact factor of 53.3. Furthermore, the three main tools; the PECARN, the CHALICE and the CATCH rules, in addition to the NEXUS II rule, were all supported by dedicated and well-funded research networks, programs, and professional groups, such as the Paediatric Emergency Care Applied Research Network for the PECARN rule, the Children’s Head Injury Algorithm for the Prediction of Important Clinical Events study group for the CHALICE rule, the Paediatric Emergency Research Canada (PERC) Head Injury Study Group for the CATCH rule, and the National Emergency X-Radiography Utilization Study II for the NEXUS II rule. There is a correlation between being supported by dedicated research programs, as a tool, and having a higher assigned grade. A summary of tools’ information, development studies indices, predictive performance and quality indicators of the 14 paediatric head injury predictive tools is presented in Table 2.

**Table 2:** Summary of tools’ information, indices, predictive performance and quality

Tool	Tool Grade	Tool Information				Study Indices			Predictive Performance		Study Quality Indicators				
		Country	Year	Citations	Studies	Citation Index	Publication Index	Literature Index	Sensitivity	Specificity	Development Method	Patient Sample Size	Number of Authors	Journal Impact	Dedicated Support
PECARN [49]	A2	USA	2009	885	24	88.5	2.40	21.24	0.97	0.59	R	42,412	32	53.25	Yes
CHALICE [43]	B2	UK	2006	309	15	23.8	1.15	4.64	0.98	0.86	R	22,772	6	3.26	Yes
CATCH [51]	C1	USA	2006	319	12	24.5	0.92	3.83	0.98	0.50	R	3866	14	6.80	Yes
NEXUS II [50, 54]	C1	USA	2005	124	6	8.9	0.43	0.74	0.99	0.15	R	1666	8	5.70	Yes
Palchak [52]	C2	USA	2003	248	3	15.5	0.19	0.74	1.00	0.46	R	2043	10	5.35	No
Haydel [47]	C3	USA	2003	118	1	7.4	0.06	0.12	1.00	0.24	R	175	5	5.35	No
Atabaki [39]	C3	USA	2008	111	1	10.1	0.09	0.11	1.00	0.46	R	1000	8	5.73	No
Buchanich [40]	C3	USA	2007	4	1	0.3	0.08	0.00	1.00	0.40	R	97	1	1.00	No
Da Dalt [41]	C0	Italy	2006	85	1	6.5	0.08	0.09	1.00	0.87	M	3806	8	1.79	No
Greenes [44, 45]	C0	USA	1999	237	2	11.9	0.10	0.47	0.53	0.72	M	422	2	5.70	No
Klemetti [48]	C0	Finland	2009	18	1	1.8	0.10	0.02	0.94	0.29	M	485	4	1.07	No
Quayle [53]	C0	USA	1997	291	1	13.2	0.05	0.29	0.44	0.85	M	322	7	5.70	No
Dietrich [42]	C0	USA	1993	220	1	8.5	0.04	0.22	1.00	0.17	M	324	5	5.35	No
Güzel [46]	C0	Turkey	2009	17	1	1.7	0.10	0.02	0.69	0.43	M	916	6	1.00	No

Citation Index (Average Annual Citations) = (number of citations/age of development study), Publication Index (Average Annual Studies) = (number of studies/age of development study), Literature Index (Citations and Publications) = (number of citations X number of studies). Age of development study = (current year – year of tool’s development). Development method: *R* Recursive Partitioning, *M* Multivariate Logistic Regression

Additional file 1: Figure S3 shows the tools’ distribution by their assigned grade. Additional file 1: Figure S4 distribution by country of development. Additional file 1: Figure S5 distribution by year of development. Additional file 1: Figure S6 number of citations of each tool. Additional file 1: Figure S7 number of studies reporting each tool. Additional file 1: Figure S8 size of patient samples used for development. Additional file 1: Figure S9 number of authors contributing to each tool. Additional file 1: Figure S10 the journal impact factor publishing each tool. Additional file 1: Figure S11 percentage of tools developed with/without dedicated support.

## Discussion

This study presents a new evidence-based approach to grade and assess predictive tools. Based on the critical appraisal of the published evidence on predictive tools, the GRASP framework uses three dimensions to grade the tools: 1) phase of evaluation; before implementation, during planning for implementation and after implementation, 2) level of evidence; adding a numerical score within each phase, and 3) direction of evidence; positive, negative or mixed. The final grade is based on the highest phase of evaluation, supported by the highest level of positive evidence, or mixed evidence that supports a positive conclusion. Among the 14 paediatric head injury predictive tools, the PECARN rule stands out clearly, since it is the only tool evaluated in post-implementation impact studies, which needs some explanation.

The 14 predictive tools targeted variable paediatric age groups. Most of the tools focused on children less than 16 years of age. However, some tools extended their cover to less than 21 years, such as Atabaki, while others limited their population to children less than 2 or 3 years, such as Buchanich and Greenes. The tools used different development methodologies and their prediction models used different mix of clinical variables. Furthermore, the predictive performances of the tools, such as their sensitivities and specificities, were different. However, the predictive performances of the tools were not correlated with their assigned grades. This indicates that the technical specifications of the predictive tools did not, in the first place, influence their successful validation, acceptance, or implementation. The country and year of tools’ development were also non-significantly influential on their successful path from validation into implementation. On the other hand, the number of citations of the studies, describing the development of the tools, and the number of studies reporting them are clearly correlated with tools’ success. These two indicators are secondary to the main quality indicators of the tools’ development studies, such as the sample size of patients used in the development of the tools and the number of researchers developing these tools.

In addition, the experiences of the researchers have an important role in leading better-quality studies. Three of the researchers who developed the PECARN rule have already contributed to older but less successful tools. Before leading the team to develop the PECARN rule in 2009, Dr. Kuppermann contributed to developing the Quayle rule in 1997 and the Palchak rule in 2003. Dr. Quayle and Dr. Atabaki each developed her own rule in 1997 and 2008, before joining the team in developing the PECARN rule in 2009. The affiliations of the researchers, to highly ranked institutes, and the support of the studies by dedicated and well-funded research networks, programs, and professional groups, added to the credibility of the tools among clinicians and organisations. As a result of the better quality and higher credibility, the PECARN rule development study was published in a top ranked journal with a high impact factor; the Lancet. In addition, the three main tools; the PECARN, the CHALICE and the CATCH rules were endorsed by professional organisations and recommended in clinical practice guidelines, such as the paediatric head trauma clinical guidelines developed by the Royal Australian and New Zealand College of Radiologists [90].

Many studies compared paediatric head injury predictive tools. Among these, nine compared the three main tools; the PECARN, the CHALICE and the CATCH rules. Despite the fact that most of the studies reported PECARN as the highest quality tool, they reported that all three predictive tools had excellent sensitivities and performed well in assessing the outcome of clinically important TBI, suggesting that all were appropriate for use in assessing mild head injury in the ED [58, 91]. However, each tool is applicable to a different proportion of children with head injury. This makes the direct comparison of the three tools difficult [72]. The CHALICE rule applies to a broad population of head injuries of any severity, the PECARN rule was developed for minor head injuries only and the CATCH rule focused on a group of patients with specific signs or symptoms [59]. The PECARN rule is the most validated [37], and has the best sensitivity while the CHALICE rule has the best specificity [66, 91, 92]. Compared to senior, experienced, and high accuracy emergency physicians, the implementation of PECARN, CATCH or CHALICE rules have a potential to increase the CT rates with limited potential to increase the accuracy of detecting clinically important TBI [93]. In addition, the three tools were not more cost-effective than usual care in some ED settings [94]. Despite that CT is the imaging modality of choice in the ED, because of availability and speed, however, magnetic resonance imaging is recently becoming the preferred modality in children. This would change predictive tools’ comparability and priority for recommendation, where further research is required [92].

Some predictive tools, in other clinical areas, gained their widespread acceptance and successful implementation by providing simplicity and feasibility. The Ottawa ankle and the Ottawa knee rules are good examples of simple paper based five items check lists, designed to exclude the need for an X-ray for possible bone fracture in adult patients at the ED [95, 96]. The resources needed to implement such tools are minimal; no technical requirements, special training or financial support are needed. Both tools were implemented, within 2 years of their development, and demonstrated positive post-implementation impact on the efficiency of ED healthcare services through wide scale high quality experimental studies [97–100].

Accordingly, selecting effective predictive tools remains a major challenge for most clinicians who usually lack the time and experience required to evaluate such tools; assessing their quality or grading their level of evidence, especially as their number and complexity have increased tremendously over the recent years. This is made worse by the complex nature of the evaluation process itself and the variability in the quality of published evidence. Furthermore, it is not always feasible to compare tools, even those designed for the same predictive tasks, due to the diversity of their development methodologies, clinical variables, target populations, conditioned applications, and predictive performances. Therefore, we chose not to look at the details of every single validation or implementation study. Alternatively, the GRASP framework provides users with a higher level and evidence-based approach to grade predictive tools through the critical appraisal of published evidence on their development and validation before implementation, usability and potential effect during planning for implementation, and post-implementation impact on clinical effectiveness, patient safety and healthcare efficiency. Based on the available evidence, the framework identifies tools that are more trusted by clinicians and researchers and consequently can be more successful. Using the GRASP framework might need some training for expert healthcare professionals and researchers, who are going to grade predictive tools and some awareness for end user clinicians who are going to use GRASP output to select predictive tools.

The main limitations of this study include the possibility of missing some predictive tools which could have been developed by clinicians but not yet published, because the GRASP framework depends on grading predictive tools based on their published evidence. Similarly, some of the published predictive tools could have been implemented in clinical practice but no studies, reporting their implementation or evaluating their post-implementation impact, have been published yet. Furthermore, while this study is in press or soon after it is published, an evidence on some tools may become available and could have an influence on the assigned grade.

## Conclusion

Comparing the three main tools, which were assigned the highest GRASP grades PECARN, CHALICE and CATCH, to the remaining 11, we find that three main factors are highly crucial and indicate better tools. Firstly, the quality of the predictive tools, which is indicated by the development methodology of the tools, the patient sample size used for development, and the number of contributing authors. The quality is also reflected through the number of citations and number of studies discussing each tool. Secondly, the experience and credibility of the tools’ authors, reflected in their clinical specialty and affiliated organisations. Thirdly, the support by dedicated and well-funded research programs. These three factors were more significantly influential, than the sole high predictive performance, on the wide acceptance and successful implementation of the tools. In addition, tools’ simplicity and feasibility, in terms of resources needed, financial support, technical requirements, complexity and number of predictors, and training, are crucial factors of their success. It is important to select tools which best fit the intended tasks, the clinical conditions, the healthcare settings and the patient populations. Based on detailed specifications, a group of best predictive tools can be recommended for use in clinical practice. Through evidence-based grading of predictive tools, the GRASP framework confirmed the PECARN rule as the highest quality tool, compared to the other tools, which have variable levels of supporting evidence. The online availability of the GRASP framework will enable clinicians and clinical guideline developers to access detailed information, reported evidence and assigned grades of predictive tools. However, keeping such information up-to-date requires continuous updating of tools’ reports when new evidence becomes available.

## Additional file

Additional file 1: **Figure S2.** Searching the literature for predictive tools and related published evidence. **Figures S3 to S11.** Statistical figures describing the fourteen paediatric head injury clinical predictive tools. **Table S3.** The GRASP Framework Detailed Report template. **Tables S4 to S17.** The GRASP Framework Detailed Report on each of the fourteen paediatric head injury clinical predictive tools. (PDF 958 kb)

## Data Availability

Data sharing is not applicable to this article as no datasets were generated or analysed during the current study.

## Abbreviations

CATCH: Canadian Assessment of Tomography for Childhood Head injury
CDC: Centers for Disease Control and Prevention
CDS: Clinical Decision Support
CHALICE: Children’s Head injury ALgorithm for the prediction of Important Clinical Events
CINAHL: Cumulative Index to Nursing and Allied Health Literature
CT: Computed Tomography
ED: Emergency Department
EMBASE: Excerpta Medica Abstract Journals Database
GRASP: Grading and Assessment of Predictive Tools for Clinical Decision Support
MEDLINE: Medical Literature Analysis and Retrieval System Online
NEXUS: National Emergency X-Radiography Utilization Study
PECARN: Paediatric Emergency Care Applied Research Network
PERC: Paediatric Emergency Research Canada
TBI: Traumatic Brain Injury
US: United States
