# Supplementary material for: Grading and assessment of clinical predictive tools for paediatric head injury: a new evidence-based approach
Source: BMC Emerg Med. 2019 Jun 14;19:35. doi: 10.1186/s12873-019-0249-y (PMC6570950; doi:10.1186/s12873-019-0249-y)
Supplement: Supplementary file 1 — Figure S2. Searching the literature for predictive tools and related published evidence. Figures S3 to S11. Statistical figures describing the fourteen paediatric head injury clinical predictive tools. Table S3. The GRASP Framework Detailed Report template. Tables S4 to S17. The GRASP Framework Detailed Report on each of the fourteen paediatric head injury clinical predictive tools. (PDF 958 kb) [file 12873_2019_249_MOESM1_ESM.pdf]

8. The Appendix

8.1. Searching the Literature for Predictive Tools and Related Published Evidence

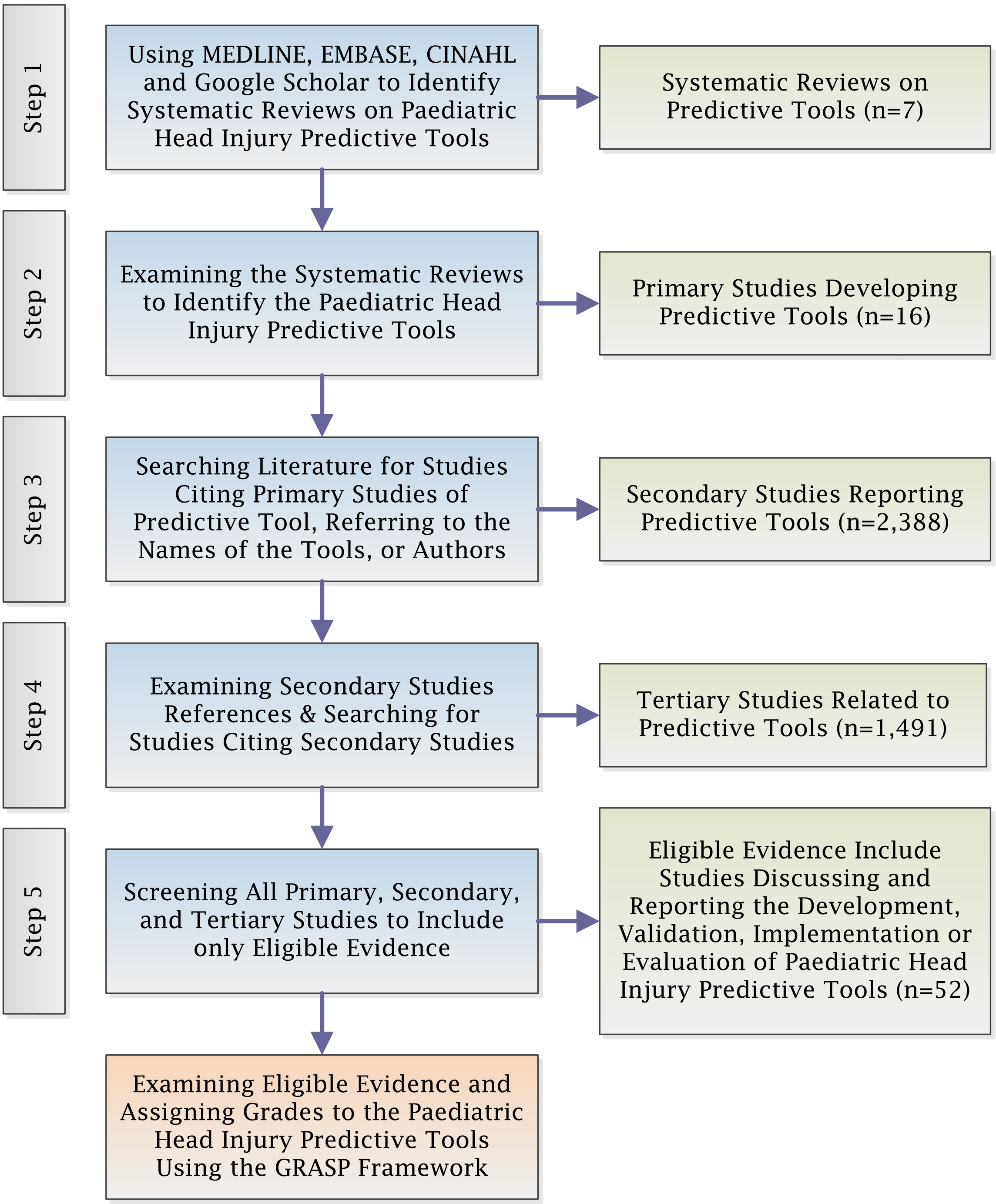

Figure 2: Searching the literature for paediatric head injury predictive tools and their related published evidence

8.2. Statistical Figures

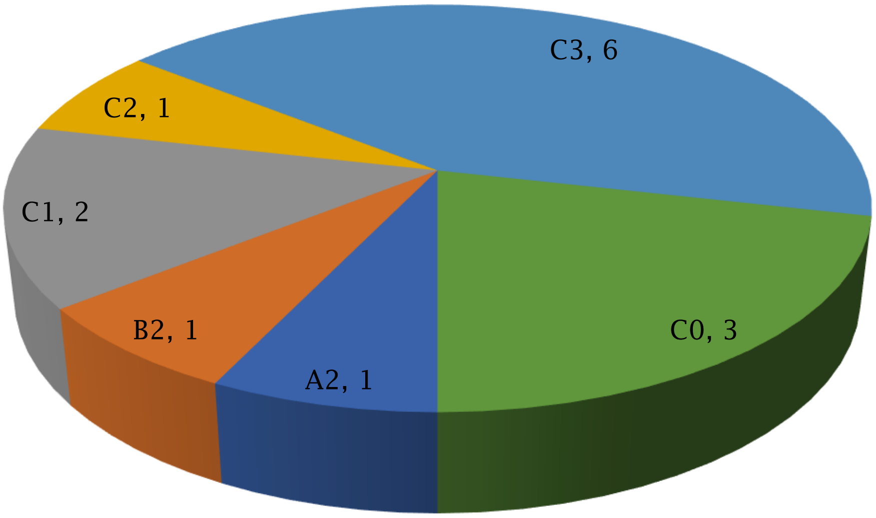

Figure 3: Tools distribution by their assigned grades
(Grade and number of tools)

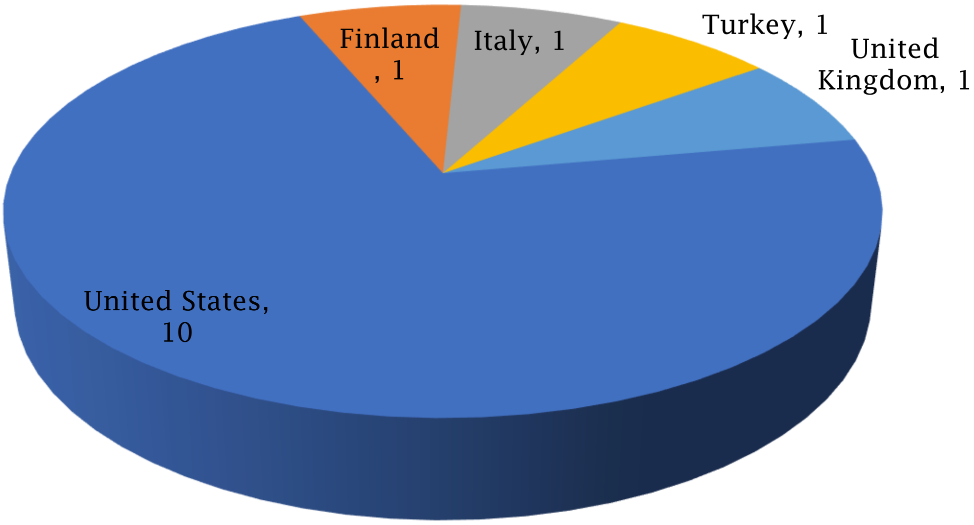

Figure 4: Tools distribution by their country of development
(Country and number of tools)

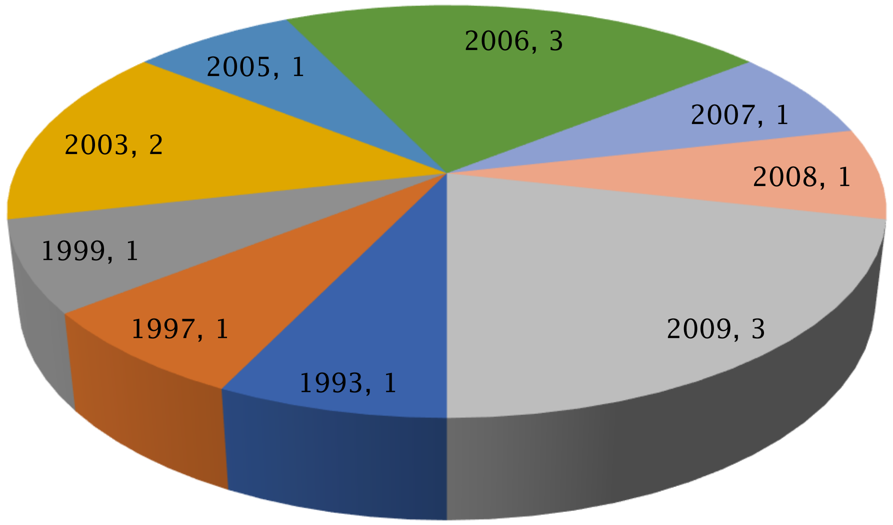

Figure 5: Tools distribution by their year of development
(Year and number of tools)

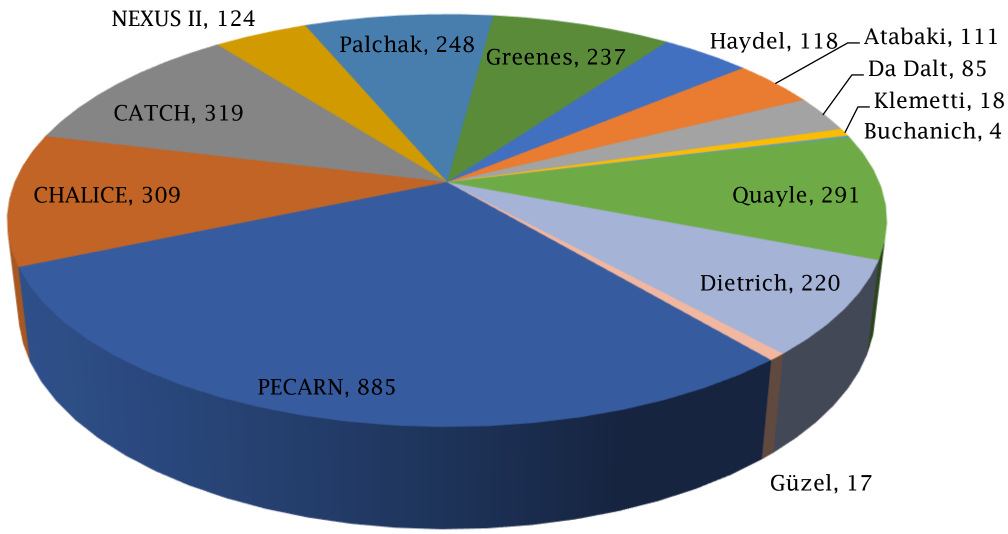

Figure 6: The number of citations of each tool

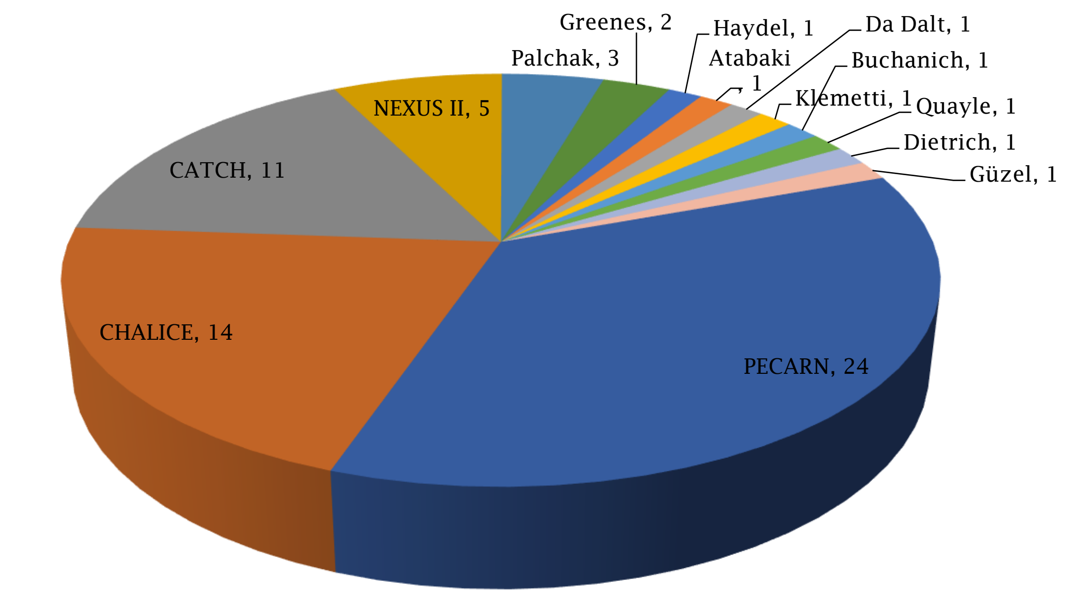

Figure 7: The number of studies reporting each tool

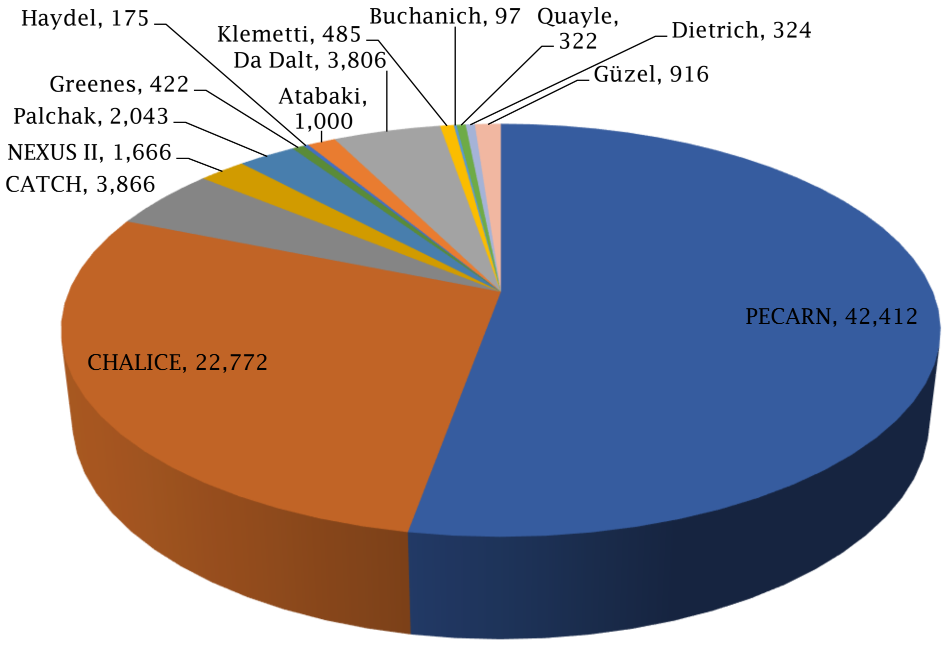

Figure 8: The size of patient samples used for developing each tool

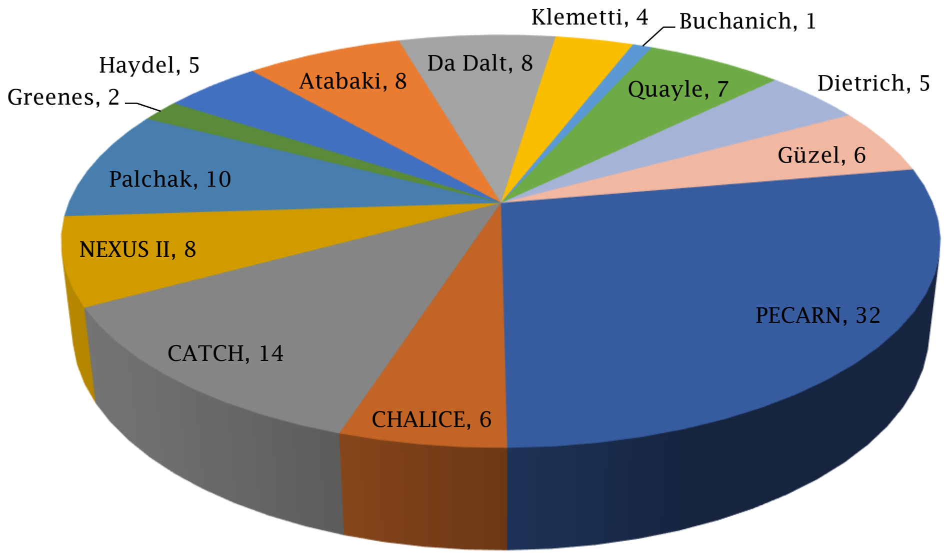

Figure 9: The number of authors contributing to the development of each tool

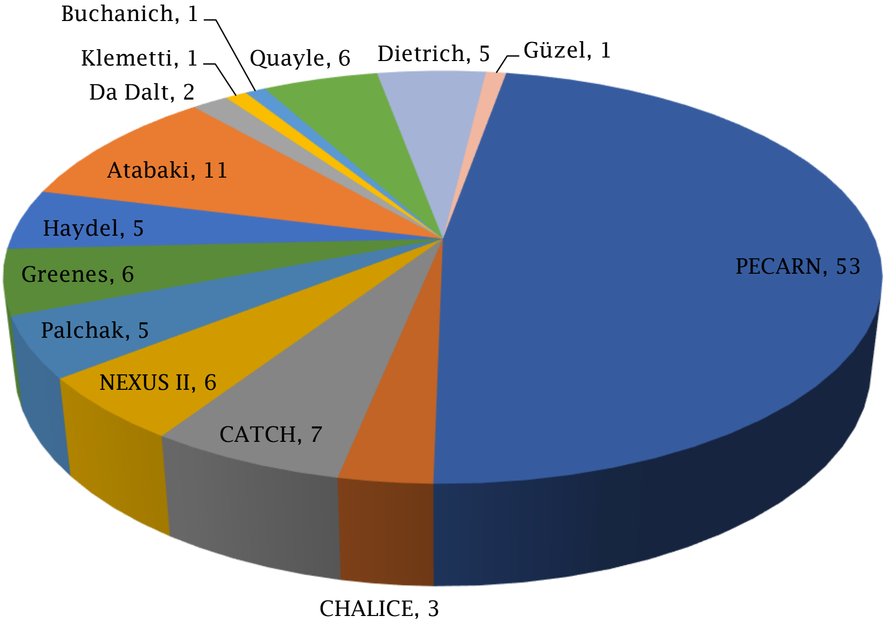

Figure 10: The journal impact factor publishing each tool

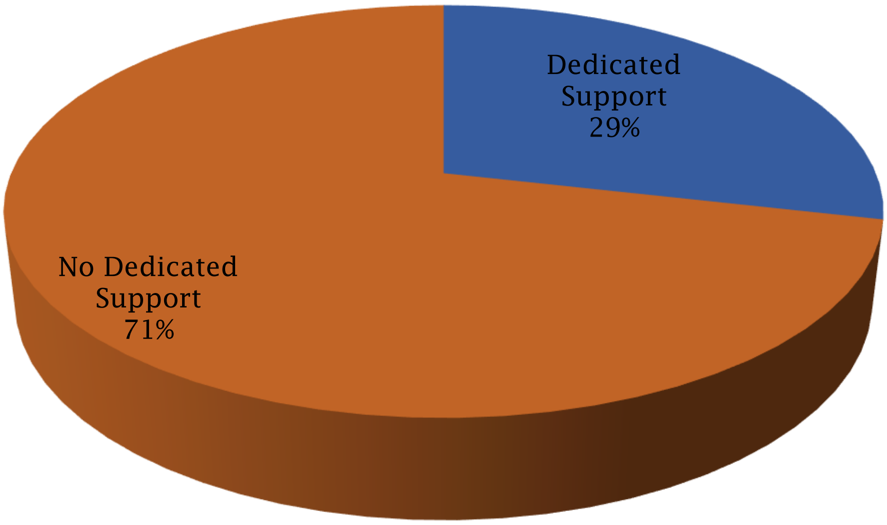

Figure 11: The percentage of tools developed with/without dedicated support

8.3. The GRASP Framework Detailed Report

Table 3: The GRASP Framework Detailed Report

**Name**	Name of predictive tool (report tool's creators and year in the absence of a given name)
**Author**	Name of developer (first author or researcher)
**Country**	Country of development
**Year**	Year of development
**Category**	Diagnostic/Therapeutic/Prognostic/Preventive
**Intended use**	Specific aim/intended use of the predictive tool
**Intended user**	Type of practitioner intended to use the tool
**Clinical area**	Clinical specialty
**Target Population**	Target patient population and health care settings in which the tool is applied
**Target Outcome**	Event to be predicted (including prediction lead time if needed)
**Action**	Recommended action based on tool's output
**Input source**	• Clinical (including Diagnostic, Genetic, Vital signs, Pathology)<br>• Non-Clinical (including Healthcare Utilisation)
**Input type**	• Objective (Measured input; from electronic systems or clinical examination)<br>• Subjective (Patient reported; history, checklist ...etc.)
**Local context**	Is the tool developed using location-specific data? (e.g. life expectancy tables)
**Methodology**	Type of algorithm used for developing the tool (e.g. parametric/non-parametric)
**Internal Validation**	Method of internal validation
**Dedicated Support**	Name of the supporting/funding research networks, programs, or professional groups
**Endorsement**	Organisations endorsing the tool and/or clinical guidelines recommending its utilisation
**Automation Flag**	Automation status (manual/automated)

**Tool Citations**	Total citations of the tool		
**Studies**	Number of studies reporting the tool		
**Authors No**	Number of authors		
**Sample Size**	Size of patient/record sample used in the development of the tool		
**Journal Name**	Name of the journal that published the tool's primary development study		
**Journal Rank**	Impact factor of the journal		
**Citation Index**	Calculated as: Average Annual Citations = number of citations/age of primary publication		
**Publication Index**	Calculated as: Average Annual Studies = number of studies/age of primary publication		
**Literature Index**	Calculated as: Citations and Publications = number of citations X number of studies		
**Phase of Evaluation**	**Level of Evidence**	**Grade**	**Evaluation Studies**
**Phase C:**<br><br>**Before implementation**<br><br>**Is it possible?**	Insufficient internal validation	**C0**	Not tested for internal validity, insufficiently internally validated, or internal validation was insufficiently reported.
	Internal validation	**C3**	Tested for internally validity (reported calibration & discrimination; sensitivity, specificity, positive and negative predictive values & other predictive performance measures).
	External validation	**C2**	Tested for external validity, using one external dataset.
	External validation multiple times	**C1**	Tested multiple times for external validity, using more than one external dataset.
**Phase B:**<br><br>**Planning for implementation**<br><br>**Is it practicable?**	Usability	**B3**	Reported usability testing (tool effectiveness, efficiency, satisfaction, learnability, memorability, and minimizing errors).
	Potential effect	**B2**	Reported estimated potential effect on clinical effectiveness, patient safety or healthcare efficiency.
	Potential effect & Usability	**B1**	Both potential effect and usability are reported.
**Phase A:**<br><br>**After implementation:**<br><br>**Is it desirable?**	Evaluation of post-implementation impact on Clinical Effectiveness, Patient Safety or Healthcare Efficiency	**A3**	Based on subjective studies; e.g. the opinion of a respected authority, clinical experience, a descriptive study, or a report of an expert committee or panel.
		**A2**	Based on observational studies; e.g. a well-designed cohort or case-control study.
		**A1**	Based on experimental studies; properly designed, widely applied randomised/nonrandomised controlled trial.
**Assigned Grade**	**Grade ABC/123**		**A1** | **A2** | **A3** | **B1** | **B2** | **B3** | **C1** | **C2** | **C3**
**Direction of Evidence**	● Positive Evidence		◕ Mixed Evidence Supporting Positive Conclusion
	○ Negative Evidence		◔ Mixed Evidence Supporting Negative Conclusion
**Justification**	Explains how the final grade is assigned based on evidence; which conclusions were taken into consideration, as positive evidence, and which were considered negative.		
**References**	Details of studies that support the justification: phase of evaluation, level of evidence, direction of evidence, study type, study settings, methodology, results, findings and conclusions (highlighted according to the findings codes).		
**Findings Codes**	Positive Findings / Negative Findings / Important Findings		

8.4. PECARN Rule – Grade A2

Table 4: The GRASP Framework Detailed Report of the PECARN Rule

**Name**	PECARN (Paediatric Emergency Care Applied Research Network) Head Injury/Trauma Rule		
**Authors/Year**	Dr. Nathan Kuppermann, United States, 2009		
**Category**	Diagnostic		
**Intended use**	Predicts need for brain imaging after paediatric head injury (Identify children who are at very low risk of clinically important brain injury).		
**Intended user**	Physicians		
**Clinical area**	Emergency department (ED)		
**Target Population**	Children less than 18 years of age at ED for head trauma		
**Target Outcome**	Traumatic brain injury		
**Action**	Do/Do Not Consider CT + Acute intervention		
**Input source**	Objective data (clinical examination) + subjective data (reported by child/parents)		
**Input type**	Clinical data: Age < or > 2 years, GCS ≤14, altered mental status, palpable skull fracture, scalp haematoma, loss of consciousness, severe injury mechanism, severe headache and history of vomiting.		
**Local context**	Input does not depend on local context of data		
**Methodology**	Recursive partitioning		
**Int. Validation**	Cross validation + Separate validation population		
**Dedicated Supp**	Paediatric Emergency Care Applied Research Network, USA.		
**Endorsement**	Recommended by:<br>• Paediatric Emergency Care Applied Research Network, a federally funded paediatric emergency medicine research network, United States.<br>• Royal Australian & New Zealand College of Radiologists, 2015 for Paediatric Head Trauma https://www.ranzcr.com/documents/3839-print-version-paediatric-head-trauma/file		
**Automation Flag**	Manually used		
**Tool Citations**	885	Reported in 24 studies	
**Authors**	32	Sample Size = 42,412	
**Journal Impact**	53.3	The Lancet	
**Phase of Evaluation**	**Level of Evidence**	**Grade**	**Evaluation Studies**
**Phase C: Before implementation Does the tool work? Is it possible?**	Internal validation	**C3**	Developed and internally validated:<br>• Kuppermann et al, 2009 (49)
	External validation	**C2**	Externally validated
	External validation multiple times	**C1**	Externally validated multiple times:<br>• Ahmadi & Yousefifard, 2017 (Systematic Review) (55):<br>○ Fuller et al, 2012 (67)<br>○ Mihindu et al, 2014 (73)<br>○ Schonfeld et al, 2014 (76)<br>○ Easter et al, 2014 (66)<br>○ Lorton et al, 2016 (71)<br>○ Atabaki et al, 2016 (56)<br>○ Babl et al, 2017 (58)<br>○ Ide et al, 2017 (70)<br>○ Nakhjavan-Shahraki et al, 2017 (74)<br>• Lyttle et al, 2013 (72)<br>• Thiam, Yap & Chong, 2015 (77)<br>• Babl & Bressan, 2015 (59)<br>• Bozan et al, 2017 (63)<br>• Babl et al, 2018 (61)
**Phase B: Planning for implementation: Is the tool practicable?**	Usability	**B3**	Not reported
	Potential effect	**B2**	Estimated potential effect:<br>• Holmes et al, 2013 (69)<br>• Nishijima et al, 2015 (75)

<table>
<tr><td rowspan="2"></td><td></td><td></td><td>Barrett, 2016 (62)
Gökharman et al, 2017 (68)</td></tr>
<tr><td>Potential effect & Usability</td><td>B1</td><td>Not Applicable</td></tr>
<tr><td rowspan="3">Phase A:
After implementation:
Is the tool desirable?</td><td rowspan="3">Evaluation of post-implementation impact on Clinical Effectiveness, Patient Safety or Healthcare Efficiency</td><td>A3</td><td>No subjective studies are reported</td></tr>
<tr><td>A2</td><td>Observational studies – negative conclusions:
Bressan et al, 2015 (65)
Observational studies – positive conclusions:
Bressan et al, 2012 (64)
Atabaki et al, 2017 (57)</td></tr>
<tr><td>A1</td><td>No experimental studies are reported</td></tr>
<tr><td>Assigned Grade</td><td colspan="2">Grade A2</td><td>A1 | ◕ | A3 | B1 | ● | B3 | ● | C2 | ●</td></tr>
<tr><td>Justification</td><td colspan="3">The PECARN rule was developed in 2009 and tested successfully for internal validity (49). The rule was tested fifteen times for external validity and proved externally valid in all the reported studies (56, 58-61, 63, 66, 67, 70-74, 76, 77). This qualifies the PECARN rule for grade C1. Four economic analysis studies discussed the positive potential effects of using the PECARN rule on lowering healthcare costs, decreasing frequency of CT scans and minimising exposure of children to harmful ionising radiation (62, 68, 69, 75). This qualifies the PECARN rule for grade B2. Three observational pre-and-post-implementation impact studies were conducted. One study concluded that the PECARN intermediate-risk predictors did not play a major role in the physicians' decision to perform a CT scan (65). However, the other two studies concluded that implementing and using the PECARN rule was associated with a statistically significant decrease in CT utilisation without safety or effectiveness problems (57, 64). Using the protocol, the mixed evidence here supports positive conclusion on the post-implementation impact of the PECARN rule. Accordingly, the final grade assigned to the PECARN rule is A2.</td></tr>
<tr><td>References</td><td colspan="3">Development and Internal Validation:
Kuppermann, N., Holmes, J. F., Dayan, P. S., Hoyle, J. D., Atabaki, S. M., Holubkov, R., ... & Badawy, M. K. (2009). Identification of children at very low risk of clinically-important brain injuries after head trauma: a prospective cohort study. The Lancet, 374(9696), 1160-1170.
External Validation:
Ahmadi, S., & Yousefifard, M. (2017). Accuracy of Pediatric Emergency Care Applied Research Network Rules in Prediction of Clinically Important Head Injuries; A Systematic Review and Meta-Analysis. International Journal of Pediatrics, 5(12), 6285-6300. Results: Data from 10 studies were included in this meta-analysis. Area under the curve (101) of SROC for PECARN model in prediction of ciTBI in children younger than 2 years old was 0.85 (95% CI: 0.82-0.88). Sensitivity, specificity and diagnostic odds ratio of this model were also calculated to be 0.98 (95% CI: 0.92-1.0), 0.56 (95% CI: 0.48-0.64) and 82.53 (95% CI: 16.23-419.63), respectively. AUC of SROC for this model in prediction of ciTBI in children aged 2-18 years old was also found to be 0.97 (95% CI: 0.95-0.98) with a sensitivity, specificity and diagnostic odds ratio of 0.98 (95% CI: 0.95-0.99), 0.60 (95% CI: 0.53-0.67) and 80.73 (95% CI: 30.59-213.05). Conclusion: The findings of this study are indicative of a high screening value for PECARN model in prediction of ciTBI and classification of patients. So it is recommended that the decision rule be used in routine practice for children referring with mild traumatic brain injuries.
Fuller, G., Dunning, J., Batchelor, J., & Lecky, F. (2012, April). An External Validation of the PECARN Clinical Decision Rule for CT Head Imaging of Infants with Minor Head Injury. In BRAIN INJURY (Vol. 26, No. 4-5, pp. 429-430). TELEPHONE HOUSE, 69-77 PAUL STREET, LONDON EC2A 4LQ, ENGLAND: INFORMA HEALTHCARE.
Mihindu, E., Bhullar, I., Tepas, J., & Kerwin, A. (2014). Computed tomography of the head in children with mild traumatic brain injury. The American surgeon, 80(9), 841-843.
Schonfeld, D., Bressan, S., Da Dalt, L., Henien, M. N., Winnett, J. A., & Nigrovic, L. E. (2014). Pediatric Emergency Care Applied Research Network head injury clinical prediction rules are reliable in practice. Archives of disease in childhood, archdischild-2013.
Easter, J. S., Bakes, K., Dhaliwal, J., Miller, M., Caruso, E., & Haukoos, J. S. (2014). Comparison of PECARN, CATCH, and CHALICE rules for children</td></tr>
</table>

with minor head injury: a prospective cohort study. Annals of emergency medicine, 64(2), 145-152.

  - Lorton, F., Poullaouec, C., Legallais, E., Simon-Pimmel, J., Chêne, M. A., Leroy, H., ... & Gras-Le Guen, C. (2016). Validation of the PECARN clinical decision rule for children with minor head trauma: a French multicenter prospective study. Scandinavian journal of trauma, resuscitation and emergency medicine, 24(1), 98.

  - Atabaki, S. M., Hoyle Jr, J. D., Schunk, J. E., Monroe, D. J., Alpern, E. R., Quayle, K. S., ... & Dayan, P. S. (2016). Comparison of prediction rules and clinician suspicion for identifying children with clinically important brain injuries after blunt head trauma. Academic emergency medicine, 23(5), 566-575.

  - Babl, F. E., Borland, M. L., Phillips, N., Kochar, A., Dalton, S., McCaskill, M., ... & Lyttle, M. D. (2017). Accuracy of PECARN, CATCH, and CHALICE head injury decision rules in children: a prospective cohort study. The Lancet.

  - Ide, K., Uematsu, S., Tetsuhara, K., Yoshimura, S., Kato, T., & Kobayashi, T. (2017). External Validation of the PECARN Head Trauma Prediction Rules in Japan. Academic Emergency Medicine, 24(3), 308-314.

  - Nakhjavan-Shahraki, B., Yousefifard, M., Hajighanbari, M. J., Oraii, A., Safari, S., & Hosseini, M. (2017). Pediatric Emergency Care Applied Research Network (PECARN) prediction rules in identifying high risk children with mild traumatic brain injury. European journal of trauma and emergency surgery, 43(6), 755-762.

- Lyttle, M. D., Cheek, J. A., Blackburn, C., Oakley, E., Ward, B., Fry, A., ... & Babl, F. E. (2013). Applicability of the CATCH, CHALICE and PECARN paediatric head injury clinical decision rules: pilot data from a single Australian centre. Emerg Med J, 30(10), 790-794. 1,012 patients (69.9%) were enrolled with 949 available for analysis. Mean age was 6.8 years (21% <2 years). 95% had initial Glasgow Coma Scale 15. CT rate was 12.8% and neurosurgery rate was 0.7%. No CDR was applicable to all patients. CHALICE was applicable to the most (97%, 95% CI 96% to 98%) and CATCH to the fewest (26%, 95% CI 24% to 29%). PECARN was applicable to 76% (95% CI 70% to 82%) aged <2 years, and 74% (95% CI 71% to 77%) aged 2–<18 years.

- Babl, F. E., & Bressan, S. (2015). Physician practice and PECARN rule outperform CATCH and CHALICE rules based on the detection of traumatic brain injury as defined by PECARN. Evidence-based medicine, 20(1), 33-34. In 1009 children, 21 had ciTBI. All were identified by the PECARN rule and physician practice. Ranked **sensitivities** were as follows: physician practice and **PECARN 100%** (95% CI 84% to 100%), physician estimates 95% (95% CI 76% to 100%), CATCH 91% (95% CI 70% to 99%) and CHALICE 84% (95% CI 60% to 97%). Ranked **specificities** were: CHALICE 85% (95% CI 82% to 87%), physician estimates 68% (95% CI 65% to 71%), **PECARN 62%** (95% CI 59% to 66%), physician practice 50% (95% CI 47% to 53%), and CATCH 44% (95% CI 41% to 47%). **Secondary outcomes included need for neurosurgical intervention with sensitivities of 100% for PECARN and physician practice** and 75% for CATCH and CHALICE.

- Thiam, D. W., Yap, S. H., & Chong, S. L. (2015). Clinical decision rules for paediatric minor head injury: are CT scans a necessary evil. Ann Acad Med Singap, 44, 335-41. The CDRs demonstrated **sensitivities** of: CATCH 100% (54.1 to 100), CHALICE 83.3% (35.9 to 99.6), **PECARN 100%** (54.1 to 100), and **specificities** of: CATCH 80.3% (77.9 to 82.5), CHALICE 76.4% (73.8 to 78.8), **PECARN high- and intermediate-risk 61.6%** (58.8 to 64.4) and **PECARN high-risk only 96.7%** (95.5 to 97.6). Conclusion: The CDRs demonstrated high accuracy in detecting children with positive CT fi ndings but direct application in areas with low rates of signifi cant traumatic brain injury (TBI) is likely to increase unnecessary CT scans ordered. Clinical observation in most cases may be a better alternative.

- Bozan, Ö., Aksel, G., Kahraman, H. A., Giritli, Ö., & Eroğlu, S. E. (2017). Comparison of PECARN and CATCH clinical decision rules in children with minor blunt head trauma. European Journal of Trauma and Emergency Surgery, 1-7. The sensitivity of PECARN was 95 (95% CI 72–100%) and specificity was 53 (95% CI 47–60%), while the sensitivity of CATCH was 48 (95% CI 25–71%) and specificity was 83 (95% CI 79–88%).

- Babl, F. E., Oakley, E., Dalziel, S. R., Borland, M. L., Phillips, N., Kochar, A., ... & Neutze, J. (2018). Accuracy of clinician practice compared with three head injury decision rules in children: a prospective cohort study. Annals of emergency medicine, 71(6),

703-710. Clinician identification of clinically important traumatic brain injury based on CT performed had a sensitivity of 158 of 160, or 98.8% (95% confidence interval [CI] 95.6% to 99.8%) and a specificity of 17,332 of 18,753, or 92.4% (95% CI 92.0% to 92.8%). **Sensitivity of PECARN for children younger than 2 years was 42 of 42 (100.0%; 95% CI 91.6% to 100.0%), and for those 2 years and older, it was 117 of 118 (99.2%; 95% CI 95.4% to 100.0%)**; for CATCH (high/medium risk), it was 147 of 160 (91.9%; 95% CI 86.5% to 95.6%); and for CHALICE, 148 of 160 (92.5%; 95% CI 87.3% to 96.1%). Conclusion: In a setting with high clinician accuracy and a low CT rate, PECARN, CATCH, or CHALICE clinical decision rules have limited potential to increase the accuracy of detecting clinically important traumat c brain injury and may increase the CT rate. In this prospective multicenter study of 18,913 children with mild head injury, clinical judgment demonstrated sensitivity similar to that of any of the 3 decision rules, as well as higher specificity than any of them. In these nationalized health care settings, clinical decision rules for paediatric head injury did not improve on clinical judgment and would likely increase CT use.

Potential Effect:

- Nishijima, D. K., Yang, Z., Urbich, M., Holmes, J. F., Zwienenberg-Lee, M., Melnikow, J., & Kuppermann, N. (2015). Cost-effectiveness of the PECARN rule in children with minor head trauma. Annals of emergency medicine, 65(1), 72-80. (PECARN strategy used fewer cranial CT scans (274 versus 353), resulted in fewer radiation-induced cancers (0.34 versus 0.45), cost less ($904,940 versus $954,420), and had lower net quality-adjusted life-year loss (-4.64 versus -5.79). PECARN strategy is more effective and less costly than usual care).

- Gökharman, F. D., AYDIN, S., Fatihoğlu, E., & KOŞAR, P. N. (2017). Pediatric Emergency Care Applied Research Network head injury prediction rules: on the basis of cost and effectiveness. Turkish journal of medical sciences, 47(6), 1770-1777. (Thus, following the PECARN rule, the treatment of 825 (79.2%) patients could be managed without cranial CT. It can be inferred from the data that unnecessary cranial CT imaging entailed a cost of approximately US $13,750–16,500 and a total X-ray dose of 1650–2062 mSv).

- Barrett, J. (2016). The Use of Clinical Decision Rules to Reduce Unnecessary Head CT Scans in Pediatric Populations (Doctoral dissertation, The University of Arizona.). (Both the CHALICE and PECARN CDRs have the potential to reduce scan rates in our home institution. The CHALICE CDR would have resulted in a greater reduction in CT scans. PECARN also would have reduced the number of scans in children 2 years and older, but not in children <2 years old).

- Holmes, M. W., Goodacre, S., Stevenson, M. D., Pandor, A., & Pickering, A. (2013). The cost-effectiveness of diagnostic management strategies for children with minor head injury. Archives of disease in childhood, 98(12), 939-944. (Our economic analysis confirms that the use of CT scanning as determined by a clinical decision rule is a cost-effective use of healthcare resources for paediatric patients).

Implementation:

- Bressan, S., Romanato, S., Mion, T., Zanconato, S., & Da Dalt, L. (2012). Implementation of adapted PECARN decision rule for children with minor head injury in the pediatric emergency department. Academic Emergency Medicine, 19(7), 801-807. (PECARN rule was successfully implemented, achieving high adherence and satisfaction of medical staff. Its use determined a low CT scan rate that was unchanged compared to previous clinical practice and showed an optimal safety and high efficacy profile. Strict monitoring is mandatory to evaluate the long-lasting benefit in patient care and/or resource utilization).

- Bressan, S., Steiner, I. P., Mion, T., Berlese, P., Romanato, S., & Da Dalt, L. (2015). The Pediatric Emergency Care Applied Research Network intermediate-risk predictors were not associated with scanning decisions for minor head injuries. Acta paediatrica, 104(1), 47-52. (The PECARN intermediate-risk predictors did not play a major role in the decision to perform a CT scan. The only factor significantly associated with the decision to perform a CT scan was when the patient was younger than 3 months of age).

- Atabaki, S. M., Jacobs, B. R., Brown, K. M., Shahzeidi, S., Heard-Garris, N. J., Chamberlain, M. B., ... & Chamberlain, J. M. (2017). Quality Improvement in Pediatric Head Trauma with PECARN rule Implementation as Computerized Decision Support. Pediatric Quality & Safety, 2(3), e019. (Statistical process control charts confirmed decreased CT rates over time POST that was not present PRE. Secondary statistical analyses confirmed that CT scan utilization rates decreased from 26.8% to 18.9%

	(unadjusted Odds Ratio [OR], 0.64; 95% Confidence Interval [CI], 0.53 -0.76; adjusted OR, 0.71; 95% CI, 0.58 -0.86). Length of stay was unchanged. There was no increase in returns within 7 days and no significant missed diagnoses).<br><br>Additional Commentary and Reviews:<br>• Maguire, J. L., Kulik, D. M., Laupacis, A., Kuppermann, N., Uleryk, E. M., & Parkin, P. C. (2011). Clinical prediction rules for children: a systematic review. Pediatrics, 128(3), e666-e677.<br>• Pickering, A., Harnan, S., Fitzgerald, P., Pandor, A., & Goodacre, S. (2011). Clinical decision rules for children with minor head injury: a systematic review. Archives of disease in childhood, 96(5), 414-421.<br>• Pandor, A., Goodacre, S., Harnan, S., Holmes, M., Pickering, A., Fitzgerald, P., ... & Stevenson, M. (2011). Diagnostic management strategies for adults and children with minor head injury: a systematic review and an economic evaluation. Health technology assessment (Winchester, England), 15(27), 1.<br>• Pandor, A., Harnan, S., Goodacre, S., Pickering, A., Fitzgerald, P., & Rees, A. (2012). Diagnostic accuracy of clinical characteristics for identifying CT abnormality after minor brain injury: a systematic review and meta-analysis. Journal of neurotrauma, 29(5), 707-718.<br>• Lyttle, M. D., Crowe, L., Oakley, E., Dunning, J., & Babl, F. E. (2012). Comparing CATCH, CHALICE and PECARN clinical decision rules for paediatric head injuries. Emerg Med J, emermed-2011.
**Colour Code**	• Important Findings<br>• Less Relevant Findings<br>• Positive Findings<br>• Negative Findings

8.5. CHALICE Rule – Grade B2

Table 5: The GRASP Framework Detailed Report of the CHALICE Rule

**Name**	CHALICE (Children's Head injury ALgorithm for the prediction of Important Clinical Events) Rule	
**Authors/Year**	Dr. Joel Dunning, United Kingdom, 2006	
**Category**	Diagnostic	
**Intended use**	Predicts death, need for neurosurgical intervention or CT abnormality in children with head trauma	
**Intended user**	Physicians	
**Clinical area**	Emergency department (ED)	
**Target Population**	Children less than 16 years of age at ED for head trauma	
**Target Outcome**	Traumatic brain injury	
**Action**	Do/Do Not Consider CT + Acute intervention	
**Input source**	Objective data (clinical examination) + subjective data (reported by child/parents)	
**Input type**	Clinical data (History, Examination, and Mechanism of Injury)	
**Local context**	Input does not depend on local context of data	
**Methodology**	Recursive partitioning	
**Int. Validation**	Cross validation	
**Dedicated Supp**	Children’s Head Injury Algorithm for the Prediction of Important Clinical Events Study Group, UK	
**Endorsement**	Recommended by:<br>• NICE Guidelines 2014 (Paediatrics) - The National Institute for Health and Care Excellence, UK (https://www.nice.org.uk/guidance/cg176/evidence/full-guideline-191719837)<br>• Royal Australian & New Zealand College of Radiologists, 2015 for Paediatric Head Trauma https://www.ranzcr.com/documents/3839-print-version-paediatric-head-trauma/file	
**Automation Flag**	Manually used	
**Tool Citations**	309	Reported in 15 studies

Authors	6	Sample Size = 22,772	
**Journal Impact**	3.26	Archives of disease in childhood	
**Phase of Evaluation**	**Level of Evidence**	**Grade**	**Evaluation Studies**
**Phase C: Before implementation Does the tool work? Is it possible?**	Internal validation	**C3**	Developed and internally validated:<br>• Dunning et al, 2006 (43)
	External validation	**C2**	Externally validated
	External validation multiple times	**C1**	Externally validated multiple times:<br>• Klemetti et al, 2009 (48)<br>• Lyttle et al, 2013 (72)<br>• Easter et al, 2014 (66)<br>• Thiam, Yap & Chong, 2015 (77)<br>• Babl et al, 2014 (60)<br>• Babl & Bressan, 2015 (59)<br>• Babl et al, 2017 (58)<br>• Babl et al, 2018 (61)
**Phase B: Planning for implementation: Is the tool practicable?**	Usability	**B3**	Not reported
	Potential effect	**B2**	Estimated potential effect – negative conclusions:<br>• Crowe, Anderson & Babl, 2010 (79)<br>• Harty & Bellis, 2010 (80)<br><br>Estimated potential effect – positive conclusions:<br>• Pandor et al, 2011 (24)<br>• Holmes et al, 2013 (69)<br>• Alali et al, 2015 (78)<br>• Barrett, 2016 (62)
	Potential effect & Usability	**B1**	Not Applicable
**Phase A: After implementation: Is the tool desirable?**	Evaluation of post-implementation impact on Clinical Effectiveness, Patient Safety or Healthcare Efficiency	**A3**	No subjective studies are reported
		**A2**	No observational studies are reported
		**A1**	No experimental studies are reported
**Assigned Grade**	**Grade B2**		A1 | A2 | A3 | B1 | ◕ | B3 | ● | C2 | ●
**Justification**	The CHALICE rule was developed in 2006 and tested successfully for internal validity (43). The rule was tested seven times for external validity and proved externally valid in all the reported studies (48, 58-60, 66, 72, 77). This qualifies the CHALICE rule for grade C1. Six cost-effectiveness studies discussed the potential effects of implementing the rule; whether it would increase or decrease the number and cost of CT scans and its potential effect on exposure of children to radiation. Two of the six studies in 2010 reported that the implementation of CHALICE rule would increase the number of CT scans performed and increase the exposure of children to radiation (79, 80). However, four subsequent studies in 2011, 2013, 2015 and 2016 reported that implementing the rule would be a cost-effective strategy to safely reduce unnecessary head CT scans (24, 62, 69, 78). Using the protocol, the mixed evidence here supports positive conclusion on the cost-effectiveness and potential effects of implementing the CHALICE rule. The rule was not evaluated for usability or post-implementation impact. Accordingly, the final grade assigned to the CHALICE rule is B2.		
**References**	Development and Internal Validation:<br>• Dunning, J., Daly, J. P., Lomas, J. P., Lecky, F., Batchelor, J., & Mackway-Jones, K. (2006). Derivation of the children's head injury algorithm for the prediction of important clinical events decision rule for head injury in children. Archives of disease in childhood, 91(11), 885-891.<br><br>External Validation:<br>• Klemetti, S., Uhari, M., Pokka, T., & Rantala, H. (2009). Evaluation of decision rules for identifying serious consequences of traumatic head injuries in pediatric patients. Pediatric emergency care, 25(12), 811-815.<br>• Lyttle, M. D., Cheek, J. A., Blackburn, C., Oakley, E., Ward, B., Fry, A., ... & Babl, F. E. (2013). Applicability of the CATCH, CHALICE and PECARN paediatric head injury clinical decision rules: pilot data from a single Australian centre. Emerg Med J, 30(10), 790-794.		

- Easter, J. S., Bakes, K., Dhaliwal, J., Miller, M., Caruso, E., & Haukoos, J. S. (2014). Comparison of PECARN, CATCH, and CHALICE rules for children with minor head injury: a prospective cohort study. Annals of emergency medicine, 64(2), 145-152.

- Thiam, D. W., Yap, S. H., & Chong, S. L. (2015). Clinical decision rules for paediatric minor head injury: are CT scans a necessary evil. Ann Acad Med Singap, 44, 335-41.

- Babl, F. E., Lyttle, M. D., Bressan, S., Borland, M., Phillips, N., Kochar, A., ... & Gilhotra, Y. (2014). A prospective observational study to assess the diagnostic accuracy of clinical decision rules for children presenting to emergency departments after head injuries (protocol): the Australasian Paediatric Head Injury Rules Study (APHIRST). BMC pediatrics, 14(1), 148.

- Babl, F. E., & Bressan, S. (2015). Physician practice and PECARN rule outperform CATCH and CHALICE rules based on the detection of traumatic brain injury as defined by PECARN. Evidence-based medicine, 20(1), 33-34.

- Babl, F. E., Borland, M. L., Phillips, N., Kochar, A., Dalton, S., McCaskill, M., ... & Lyttle, M. D. (2017). Accuracy of PECARN, CATCH, and CHALICE head injury decision rules in children: a prospective cohort study. The Lancet.

- Babl, F. E., Oakley, E., Dalziel, S. R., Borland, M. L., Phillips, N., Kochar, A., ... & Neutze, J. (2018). Accuracy of clinician practice compared with three head injury decision rules in children: a prospective cohort study. Annals of emergency medicine, 71(6), 703-710.

Potential Effect (Negative conclusions):

- Crowe, L., Anderson, V., & Babl, F. E. (2010). Application of the CHALICE clinical prediction rule for intracranial injury in children outside the UK: impact on head CT rate. Archives of disease in childhood, archdischild174854. (Implementation of the CHALICE clinical prediction rule would cause an increase in the number of CT scans. Although the CHALICE rule would have identified a very small number of additional cases with abnormal CT scans, based on our clinical set-up the majority of CT scans would have been unnecessary with resultant radiation exposure and the possible need for sedation of the child. The value of the CHALICE rule is acknowledged, but the role of expectant observation and senior staff review needs to be clarified).

- Harty, E., & Bellis, F. (2010). CHALICE head injury rule: an implementation study. Emergency medicine journal, emj-2009. (If the pre-existing (2003) guideline had been strictly applied, 28 (6%) of the 464 patients analysed would have received a computed tomography (CT) scan. Applying the 2007 guideline (based on CHALICE head injury rule) to the same 464 patients resulted in an extra 21 (4.6%) scans).

Potential Effect (Positive conclusions):

- Pandor, A., Goodacre, S., Harnan, S., Holmes, M., Pickering, A., Fitzgerald, P., ... & Stevenson, M. (2011). Diagnostic management strategies for adults and children with minor head injury: a systematic review and an economic evaluation. Health technology assessment (Winchester, England), 15(27), 1. (The CHALICE rule was the most cost-effective strategy when derivation data were used, but the NEXUS II rule was optimal where validation data were used).

- Holmes, M. W., Goodacre, S., Stevenson, M. D., Pandor, A., & Pickering, A. (2013). The cost-effectiveness of diagnostic management strategies for children with minor head injury. Archives of disease in childhood, 98(12), 939-944. (Our economic analysis confirms that the use of CT scanning as determined by a clinical decision rule is a cost-effective use of healthcare resources for paediatric patients).

- Alali, A. S., Burton, K., Fowler, R. A., Naimark, D. M., Scales, D. C., Mainprize, T. G., & Nathens, A. B. (2015). Economic evaluations in the diagnosis and management of traumatic brain injury: a systematic review and analysis of quality. Value in Health, 18(5), 721-734. (Current evidence from high-quality studies supports the economic attractiveness of a low medical threshold for CT scanning of asymptomatic infants with possible inflicted TBI, the utilization of the Canadian CT Head Rule in adults and the CHALICE rule in children as the diagnostic strategies for mild TBI).

- Barrett, J. (2016). The Use of Clinical Decision Rules to Reduce Unnecessary Head CT Scans in Pediatric Populations (Doctoral dissertation, The University of Arizona.). (Both the CHALICE and PECARN CDRs have the potential to reduce scan rates in our home institution. The CHALICE CDR would have resulted in a greater reduction in CT

	scans. PECARN also would have reduced the number of scans in children 2 years and older, but not in children <2 years old).<br><br>Additional Commentary and Reviews:<br><br>• Maguire, J. L., Boutis, K., Uleryk, E. M., Laupacis, A., & Parkin, P. C. (2009). Should a head-injured child receive a head CT scan? A systematic review of clinical prediction rules. Pediatrics, 124(1), e145-e154.<br><br>• Maguire, J. L., Kulik, D. M., Laupacis, A., Kuppermann, N., Uleryk, E. M., & Parkin, P. C. (2011). Clinical prediction rules for children: a systematic review. Pediatrics, 128(3), e666-e677.<br><br>• Pickering, A., Harnan, S., Fitzgerald, P., Pandor, A., & Goodacre, S. (2011). Clinical decision rules for children with minor head injury: a systematic review. Archives of disease in childhood, 96(5), 414-421.<br><br>• Pandor, A., Goodacre, S., Harnan, S., Holmes, M., Pickering, A., Fitzgerald, P., ... & Stevenson, M. (2011). Diagnostic management strategies for adults and children with minor head injury: a systematic review and an economic evaluation. Health technology assessment (Winchester, England), 15(27), 1.<br><br>• Pandor, A., Harnan, S., Goodacre, S., Pickering, A., Fitzgerald, P., & Rees, A. (2012). Diagnostic accuracy of clinical characteristics for identifying CT abnormality after minor brain injury: a systematic review and meta-analysis. Journal of neurotrauma, 29(5), 707-718.<br><br>• Lyttle, M. D., Crowe, L., Oakley, E., Dunning, J., & Babl, F. E. (2012). Comparing CATCH, CHALICE and PECARN clinical decision rules for paediatric head injuries. Emerg Med J, emermed-2011.<br><br>• Sempértegui Cárdenas, P. X. (2016). Validación de una escala de predicción de lesiones intracraneales para trauma cráneo-encefálico en niños de 0 a 5 años del Hospital Vicente Corral Moscoso Enero-Diciembre 2014. Estudio de test diagnóstico (Master's thesis).
**Colour Code**	• Important Findings<br>• Less Relevant Findings<br>• Positive Findings<br>• Negative Findings

8.6. CATCH Rule – Grade C1

Table 6: The GRASP Framework Detailed Report of the CATCH Rule

**Name**	CATCH Rule (Canadian Assessment of Tomography for Childhood Head injury)
**Authors/Year**	Dr. Martin Osmond, United States, 2010
**Category**	Diagnostic
**Intended use**	Predicts clinically significant head injuries in children after minor head trauma
**Intended user**	Physicians
**Clinical area**	Emergency department (ED)
**Target Population**	Children less than 16 years of age at ED for head trauma
**Target Outcome**	Traumatic brain injury
**Action**	Do/Do Not Consider CT + Acute intervention
**Input source**	Objective data (clinical examination) + subjective data (reported by child/parents)
**Input type**	Clinical data: GCS <15 at 2 hrs after injury, suspected open or depressed skull fracture, history of worsening headache, irritability on exam, any sign of basal skull fracture (hemotympanum, raccoon eyes, CSF otorrhea or rhinorrhoea, Battle's sign), large boggy scalp hematoma, dangerous mechanism of injury (MVC, fall from ≥3 ft (91 cm) or 5 stairs, fall from bicycle with no helmet).
**Local context**	Input does not depend on local context of data
**Methodology**	Recursive partitioning
**Int. Validation**	Bootstrapping method

**Dedicated Supp**	Paediatric Emergency Research Canada (PERC) Head Injury Study Group, Canada		
**Endorsement**	Recommended by the Royal Australian & New Zealand College of Radiologists, 2015 for Paediatric Head Trauma: https://www.ranzcr.com/documents/3839-print-version-paediatric-head-trauma/file		
**Automation Flag**	Manually used		
**Tool Citations**	319	Reported in 12 studies	
**Authors**	14	Sample Size = 3,866	
**Journal Impact**	6.8	Canadian Medical Association Journal	
**Phase of Evaluation**	**Level of Evidence**	**Grade**	**Evaluation Studies**
**Phase C: Before implementation Does the tool work? Is it possible?**	Internal validation	**C3**	Developed and internally validated:<br>• Osmond & Stiell, 2002 (82)<br>• Osmond et al, 2006 (83)<br>• Osmond et al, 2010 (51)
	External validation	**C2**	Externally validated
	External validation multiple times	**C1**	Externally validated multiple times:<br>• Gerdung, Dowling & Lang, 2012 (81)<br>• Klement et al, 2012 (48)<br>• Lyttle et al, 2013 (72)<br>• Easter et al, 2014 (66)<br>• Babl et al, 2014 (60)<br>• Babl & Bressan, 2015 (59)<br>• Babl et al, 2017 (58)<br>• Bozan et al, 2017 (63)<br>• Babl et al, 2018 (61)
**Phase B: Planning for implementation: Is the tool practicable?**	Usability	**B3**	Not reported
	Potential effect	**B2**	Not reported
	Potential effect & Usability	**B1**	Not reported
**Phase A: After implementation: Is the tool desirable?**	Evaluation of post-implementation impact on Clinical Effectiveness, Patient Safety or Healthcare Efficiency	**A3**	No subjective studies are reported
		**A2**	No observational studies are reported
		**A1**	No experimental studies are reported
**Assigned Grade**	**Grade C1**		A1 A2 A3 B1 B2 B3 ● C2 ●
**Justification**	The CATCH rule was developed in 2010 and tested successfully for internal validity (51). The rule was tested eight times for external validity and proved externally valid in all the reported studies (48, 58-60, 63, 66, 72, 81). The rule was not evaluated for usability, potential effect or post-implementation impact. Accordingly, the final grade assigned to the CATCH rule is C1.		
**References**	Development and Internal Validation:<br>• Osmond, M. H., & Stiell, I. G. (2002). Canadian assessment of tomography for childhood head injuries. University of Ottawa, Trauma Division of Pediatric Emergency Medicine Children's Hospital of Eastern Ontario. Personal communication.<br>• Osmond, M. H., Klassen, T. P., Stiell, I. G., & Correll, R. (2006). The CATCH rule: a clinical decision rule for the use of computed tomography of the head in children with minor head injury. Academic Emergency Medicine, 13(5 Supplement 1), S11.<br>• Osmond, M. H., Klassen, T. P., Wells, G. A., Correll, R., Jarvis, A., Joubert, G., ... & Nijssen-Jordan, C. (2010). CATCH: a clinical decision rule for the use of computed tomography in children with minor head injury. Canadian Medical Association Journal, 182(4), 341-348.<br>External Validation:<br>• Gerdung, C., Dowling, S., & Lang, E. (2012). Review of the CATCH study a clinical decision rule for the use of computed tomography in children with minor head injury. Canadian Journal of Emergency Medicine, 14(4), 247-251.		

- Klement, W., Wilk, S., Michalowski, W., Farion, K. J., Osmond, M. H., & Verter, V. (2012). Predicting the need for CT imaging in children with minor head injury using an ensemble of Naive Bayes classifiers. Artificial intelligence in medicine, 54(3), 163-170. (We showed that the proposed ensemble model achieved a more balanced predictive performance than the CATCH rule with an average sensitivity of 82.8% and an average specificity of 74.4% (vs. 98.1% and 50.0% for the CATCH rule respectively).

- Lyttle, M. D., Cheek, J. A., Blackburn, C., Oakley, E., Ward, B., Fry, A., ... & Babl, F. E. (2013). Applicability of the CATCH, CHALICE and PECARN paediatric head injury clinical decision rules: pilot data from a single Australian centre. Emerg Med J, 30(10), 790-794.

- Easter, J. S., Bakes, K., Dhaliwal, J., Miller, M., Caruso, E., & Haukoos, J. S. (2014). Comparison of PECARN, CATCH, and CHALICE rules for children with minor head injury: a prospective cohort study. Annals of emergency medicine, 64(2), 145-152.

- Babl, F. E., Lyttle, M. D., Bressan, S., Borland, M., Phillips, N., Kochar, A., ... & Gilhotra, Y. (2014). A prospective observational study to assess the diagnostic accuracy of clinical decision rules for children presenting to emergency departments after head injuries (protocol): the Australasian Paediatric Head Injury Rules Study (APHIRST). BMC pediatrics, 14(1), 148.

- Babl, F. E., & Bressan, S. (2015). Physician practice and PECARN rule outperform CATCH and CHALICE rules based on the detection of traumatic brain injury as defined by PECARN. Evidence-based medicine, 20(1), 33-34.

- Babl, F. E., Borland, M. L., Phillips, N., Kochar, A., Dalton, S., McCaskill, M., ... & Lyttle, M. D. (2017). Accuracy of PECARN, CATCH, and CHALICE head injury decision rules in children: a prospective cohort study. The Lancet.

- Bozan, Ö., Aksel, G., Kahraman, H. A., Giritli, Ö., & Eroğlu, S. E. (2017). Comparison of PECARN and CATCH clinical decision rules in children with minor blunt head trauma. European Journal of Trauma and Emergency Surgery, 1-7.

- Babl, F. E., Oakley, E., Dalziel, S. R., Borland, M. L., Phillips, N., Kochar, A., ... & Neutze, J. (2018). Accuracy of clinician practice compared with three head injury decision rules in children: a prospective cohort study. Annals of emergency medicine, 71(6), 703-710.

Additional Commentary and Reviews:

- Maguire, J. L., Kulik, D. M., Laupacis, A., Kuppermann, N., Uleryk, E. M., & Parkin, P. C. (2011). Clinical prediction rules for children: a systematic review. Pediatrics, 128(3), e666-e677.

- Pickering, A., Harnan, S., Fitzgerald, P., Pandor, A., & Goodacre, S. (2011). Clinical decision rules for children with minor head injury: a systematic review. Archives of disease in childhood, 96(5), 414-421.

- Pandor, A., Goodacre, S., Harnan, S., Holmes, M., Pickering, A., Fitzgerald, P., ... & Stevenson, M. (2011). Diagnostic management strategies for adults and children with minor head injury: a systematic review and an economic evaluation. Health technology assessment (Winchester, England), 15(27), 1.

- Lyttle, M. D., Crowe, L., Oakley, E., Dunning, J., & Babl, F. E. (2012). Comparing CATCH, CHALICE and PECARN clinical decision rules for paediatric head injuries. Emerg Med J, emermed-2011.

**Colour Code**	• Important Findings<br>• Less Relevant Findings	• Positive Findings<br>• Negative Findings

8.7. NEXUS II Rule – Grade C1

Table 7: The GRASP Framework Detailed Report of the NEXUS II Rule

**Name**	NEXUS II Rule for Adult/Paediatric Head Injury/Trauma		
**Authors/Year**	Dr. William R. Mower, United States, 2005 (designed the rule for adults) – Dr. Jennifer A Oman, United States, 2006 (validated the rule for paediatrics).		
**Category**	Diagnostic		
**Intended use**	Predict the need for computed tomography among children with head trauma		
**Intended user**	Physicians		
**Clinical area**	Emergency department (ED)		
**Target Population**	Children less than 18 years of age at ED for head trauma		
**Target Outcome**	Traumatic brain injury		
**Action**	Do/Do Not Consider CT + Acute intervention		
**Input source**	Objective data (clinical examination) + subjective data (reported by child/parents)		
**Input type**	Clinical data: Spontaneous eye opening, Orientation, Ability to follow commands, Seizure after trauma, Loss of consciousness, Prolonged loss of consciousness, Severe or progressive headache, Coagulopathy, Abnormal behaviour, Abnormal level of alertness, Evidence of significant skull fracture, Persistent vomiting, Evidence of intoxication, Motor deficit, Gait abnormality, Abnormal cerebellar function, Cranial nerve abnormality, Inability to read or write, Scalp hematoma, Neurologic deficit.		
**Local context**	Input does not depend on local context of data		
**Methodology**	Recursive partitioning		
**Int. Validation**	Cross validation		
**Dedicated Supp**	National Emergency X-Radiography Utilization Study II for the NEXUS II rule, USA.		
**Endorsement**	Not recommended by clinical guidelines		
**Automation Flag**	Manually used		
**Tool Citations**	124	Reported for paediatric head injury in 6 studies	
**Authors**	8	Sample Size = 1,666	
**Journal Impact**	5.7	Paediatrics	
**Phase of Evaluation**	**Level of Evidence**	**Grade**	**Evaluation Studies**
**Phase C: Before implementation Does the tool work? Is it possible?**	Internal validation	**C3**	Developed and internally validated for adults:<br>• Mower et al, 2002 (88)<br>• Mower et al, 2005 (89)
	External validation	**C2**	Externally validated for paediatrics
	External validation multiple times	**C1**	Externally validated for paediatrics:<br>• Oman et al, 2006 (50)<br>• Sun, Hoffman & Mower, 2007 (54)<br>• Klemetti et al, 2009 (48)<br>• Stein et al, 2009 (86)<br>• Schachar et al, 2011 (85)<br>• Gupta et al, 2018 (84)
**Phase B: Planning for implementation: Is the tool practicable?**	Usability	**B3**	Not reported
	Potential effect	**B2**	Not reported
	Potential effect & Usability	**B1**	Not reported
**Phase A: After implementation: Is the tool desirable?**	Evaluation of post-implementation impact on Clinical Effectiveness, Patient Safety or Healthcare Efficiency	**A3**	No subjective studies are reported
		**A2**	No observational studies are reported
		**A1**	No experimental studies are reported
**Assigned Grade**	**Grade C1**		A1 A2 A3 B1 B2 B3 ◕ C2 ●

**Justification**	The NEXUS II rule was developed in 2005 primarily for the diagnosis of adult head injury (88, 89). Later on, the rule was validated for paediatrics (50). The tool was then tested, four times, for external validity. One study failed to properly evaluate the rule after using a modified version, which did not show external validity (54). Two studies proved the rule was externally valid for children less than 14 and 16 years (48, 85) and one study proved the rule was externally valid for children over 10 years (86). Using the protocol, the mixed evidence here supports positive conclusion on external validity. The rule was not evaluated for usability, potential effect or post-implementation impact. Accordingly, the final grade assigned to the NEXUS II rule is C1.
**References**	Development and Internal Validation for Adults:<br><br>• Mower, W. R., Hoffman, J. R., Herbert, M., Wolfson, A. B., Pollack Jr, C. V., Zucker, M. I., & NEXUS II Investigators. (2002). Developing a clinical decision instrument to rule out intracranial injuries in patients with minor head trauma: methodology of the NEXUS II investigation. Annals of emergency medicine, 40(5), 505-515.<br><br>• Mower, W. R., Hoffman, J. R., Herbert, M., Wolfson, A. B., Pollack Jr, C. V., Zucker, M. I., & NEXUS II Investigators. (2005). Developing a decision instrument to guide computed tomographic imaging of blunt head injury patients. Journal of Trauma and Acute Care Surgery, 59(4), 954-959.<br><br>Externally Validated for Paediatrics – Positive Conclusions:<br><br>• Oman, J. A., Cooper, R. J., Holmes, J. F., Viccellio, P., Nyce, A., Ross, S. E., ... & Mower, W. R. (2006). Predictive performance of a decision rule to predict need for computed tomography among children with blunt head trauma. Pediatrics, 117(2), e238-e246. An analysis was conducted of the pediatric cohort involved in the derivation set of National Emergency X-Radiography Utilization Study II (NEXUS II). We determined the test performance characteristics of the 8-variable NEXUS II decision instrument, derived from the entire NEXUS II cohort, in the pediatric cohort (0–18 years of age), as well as in the very young children (<3 years). The decision instrument derived in the large NEXUS II cohort performed with similarly high sensitivity among the subgroup of children who were included in this study. Clinically important ICI were rare in children who did not exhibit at least 1 of the NEXUS II risk criteria.<br><br>• Sun, B. C., Hoffman, J. R., & Mower, W. R. (2007). Evaluation of a modified prediction instrument to identify significant pediatric intracranial injury after blunt head trauma. Annals of emergency medicine, 49(3), 325-332. In the NEXUS II cohort, a modified version of the University of California-Davis Rule misclassified a substantial proportion of paediatric patients with clinically important blunt head injury. Although we cannot evaluate the exact University of California-Davis Rule, we demonstrate that using stricter definitions of “headache” and “vomiting” and different wording than in the original study may have unintended or negative consequences. We emphasize the importance of careful attention to precise definitions of clinical predictors when a decision instrument is used.<br><br>• Schachar, J. L., Zampolin, R. L., Miller, T. S., Farinhas, J. M., Freeman, K., & Taragin, B. H. (2011). External validation of the New Orleans Criteria (NOC), the Canadian CT Head Rule (CCHR) and the National Emergency X-Radiography Utilization Study II (NEXUS II) for CT scanning in pediatric patients with minor head injury in a non-trauma center. Pediatric radiology, 41(8), 971.<br><br>• Gupta, M., Mower, W. R., Rodriguez, R. M., & Hendey, G. W. (2018). Validation of the Pediatric NEXUS II Head Computed Tomography Decision Instrument for Selective Imaging of Pediatric Patients with Blunt Head Trauma. Academic Emergency Medicine.<br><br>Externally Validated for Paediatrics – Equivocal and Negative Conclusions:<br><br>• Stein, S. C., Fabbri, A., Servadei, F., & Glick, H. A. (2009). A critical comparison of clinical decision instruments for computed tomographic scanning in mild closed traumatic brain injury in adolescents and adults. Annals of emergency medicine, 53(2), 180-188. NEXUS-II and the Scandinavian clinical decision aids displayed the best combination of sensitivity and specificity in this patient population (patients aged 10 years or older).<br><br>• Klemetti, S., Uhari, M., Pokka, T., & Rantala, H. (2009). Evaluation of decision rules for identifying serious consequences of traumatic head injuries in pediatric patients. Pediatric emergency care, 25(12), 811-815. We found NEXUS II to be the best of the rules tested here.<br><br>Systematic review studies:

	• Maguire, J. L., Boutis, K., Uleryk, E. M., Laupacis, A., & Parkin, P. C. (2009). Should a head-injured child receive a head CT scan? A systematic review of clinical prediction rules. Pediatrics, 124(1), e145-e154.<br>• Maguire, J. L., Kulik, D. M., Laupacis, A., Kuppermann, N., Uleryk, E. M., & Parkin, P. C. (2011). Clinical prediction rules for children: a systematic review. Pediatrics, 128(3), e666-e677.<br>• Pickering, A., Harnan, S., Fitzgerald, P., Pandor, A., & Goodacre, S. (2011). Clinical decision rules for children with minor head injury: a systematic review. Archives of disease in childhood, 96(5), 414-421.<br>• Pandor, A., Goodacre, S., Harnan, S., Holmes, M., Pickering, A., Fitzgerald, P., ... & Stevenson, M. (2011). Diagnostic management strategies for adults and children with minor head injury: a systematic review and an economic evaluation. Health technology assessment (Winchester, England), 15(27), 1.<br>• Pandor, A., Harnan, S., Goodacre, S., Pickering, A., Fitzgerald, P., & Rees, A. (2012). Diagnostic accuracy of clinical characteristics for identifying CT abnormality after minor brain injury: a systematic review and meta-analysis. Journal of neurotrauma, 29(5), 707-718.<br>• Sempértegui Cárdenas, P. X. (2016). Validación de una escala de predicción de lesiones intracraneales para trauma cráneo-encefálico en niños de 0 a 5 años del Hospital Vicente Corral Moscoso Enero-Diciembre 2014. Estudio de test diagnóstico (Master's thesis).	
**Colour Code**	• Important Findings<br>• Less Relevant Findings	• Positive Findings<br>• Negative Findings

8.8. Palchak Rule – Grade C2

Table 8: The GRASP Framework Detailed Report of Palchak Rule

**Name**	Palchak (UC Davis) Rule for Paediatric Head Injury/Trauma		
**Authors/Year**	Dr. Michael Palchak and Dr. Nathan Kuppermann, United States, 2003		
**Category**	Diagnostic		
**Intended use**	Identifies children at low risk for brain injuries after head trauma		
**Intended user**	Physicians		
**Clinical area**	Emergency department (ED)		
**Target Population**	Children less than 18 years of age at ED for head trauma		
**Target Outcome**	Traumatic brain injury		
**Action**	Do/Do Not Consider CT + Acute intervention		
**Input source**	Objective data (clinical examination) + subjective data (reported by child/parents)		
**Input type**	Clinical data: Abnormal mental status, clinical signs of skull fracture, scalp hematoma in a child ≤2 y, history of vomiting and headache.		
**Local context**	Input does not depend on local context of data		
**Methodology**	Recursive partitioning		
**Int. Validation**	Cross validation		
**Dedicated Supp**	Not supported by any research networks, programs, or professional groups.		
**Endorsement**	Not recommended by clinical guidelines		
**Automation Flag**	Manually used		
**Tool Citations**	248	Reported in 3 studies	
**Authors**	10	Sample Size = 2,043	
**Journal Impact**	5.35	Annals of emergency medicine	
**Phase of Evaluation**	**Level of Evidence**	**Grade**	**Evaluation Studies**
**Phase C:**<br>**Before implementation**	Internal validation	**C3**	Developed and internally validated:<br>• Palchak et al, 2003 (52)<br>• Palchak, Holmes & Kuppermann, 2009 (87)

**Does the tool work? Is it possible?**	External validation	**C2**	External validation:<br>• Klemetti et al, 2009 (48)
	External validation multiple times	**C1**	Not reported
**Phase B:<br>Planning for implementation:<br>Is the tool practicable?**	Usability	**B3**	Not reported
	Potential effect	**B2**	Not reported
	Potential effect & Usability	**B1**	Not reported
**Phase A:<br>After implementation:<br>Is the tool desirable?**	Evaluation of post-implementation impact on Clinical Effectiveness, Patient Safety or Healthcare Efficiency	**A3**	No subjective studies are reported
		**A2**	No observational studies are reported
		**A1**	No experimental studies are reported
**Assigned Grade**	**Grade C2**		A1 | A2 | A3 | B1 | B2 | B3 | C1 | ● | ●
**Justification**	Palchak rule was developed in 2003 and tested successfully for internal validity (52). A study by the same authors in 2009 included validation of the rule in comparison to clinician judgement using the same dataset that was used for the rule development; this is still considered an internal validation (87). One external validation study reported the predictive performance of Palchak rule was acceptable (48). The rule was not evaluated for usability, potential effect or post-implementation impact. Accordingly, the final grade assigned to Palchak rule is C2.		
**References**	Development and Internal Validation:<br>• Palchak, M. J., Holmes, J. F., Vance, C. W., Gelber, R. E., Schauer, B. A., Harrison, M. J., ... & Kuppermann, N. (2003). A decision rule for identifying children at low risk for brain injuries after blunt head trauma. Annals of emergency medicine, 42(4), 492-506.<br>• Palchak, M. J., Holmes, J. F., & Kuppermann, N. (2009). Clinician judgment versus a decision rule for identifying children at risk of traumatic brain injury on computed tomography after blunt head trauma. Pediatric emergency care, 25(2), 61-65.<br>External validation:<br>• Klemetti, S., Uhari, M., Pokka, T., & Rantala, H. (2009). Evaluation of decision rules for identifying serious consequences of traumatic head injuries in pediatric patients. Pediatric emergency care, 25(12), 811-815.<br>Additional Commentary and Reviews:<br>• Maguire, J. L., Boutis, K., Uleryk, E. M., Laupacis, A., & Parkin, P. C. (2009). Should a head-injured child receive a head CT scan? A systematic review of clinical prediction rules. Pediatrics, 124(1), e145-e154.<br>• Maguire, J. L., Kulik, D. M., Laupacis, A., Kuppermann, N., Uleryk, E. M., & Parkin, P. C. (2011). Clinical prediction rules for children: a systematic review. Pediatrics, 128(3), e666-e677.<br>• Pickering, A., Harnan, S., Fitzgerald, P., Pandor, A., & Goodacre, S. (2011). Clinical decision rules for children with minor head injury: a systematic review. Archives of disease in childhood, 96(5), 414-421.<br>• Pandor, A., Goodacre, S., Harnan, S., Holmes, M., Pickering, A., Fitzgerald, P., ... & Stevenson, M. (2011). Diagnostic management strategies for adults and children with minor head injury: a systematic review and an economic evaluation. Health technology assessment (Winchester, England), 15(27), 1.<br>• Sempértegui Cárdenas, P. X. (2016). Validación de una escala de predicción de lesiones intracraneales para trauma cráneo-encefálico en niños de 0 a 5 años del Hospital Vicente Corral Moscoso Enero-Diciembre 2014. Estudio de test diagnóstico (Master's thesis).		
**Colour Code**	• Important Findings<br>• Less Relevant Findings		• Positive Findings<br>• Negative Findings

8.9. Haydel Rule – Grade C3

Table 9: The GRASP Framework Detailed Report of Haydel Rule

**Name**	Haydel Rule for Paediatrics Head Injury/Trauma		
**Authors/Year**	Dr. Micelle J. Haydel, United States, 2003		
**Category**	Diagnostic		
**Intended use**	Identifies children at low risk for traumatic brain injuries after head trauma		
**Intended user**	Physicians		
**Clinical area**	Emergency department (ED)		
**Target Population**	Children aged 5 to 17 years at ED for head trauma		
**Target Outcome**	Traumatic brain injury		
**Action**	Do/Do Not Consider CT + Acute intervention		
**Input source**	Objective data (clinical examination) + subjective data (reported by child/parents)		
**Input type**	Clinical data: scalp hematoma, scalp abrasion, scalp laceration, forehead contusion, headache, vomiting, short-term memory deficit.		
**Local context**	Input does not depend on local context of data		
**Methodology**	Recursive partitioning		
**Int. Validation**	Separate validation population		
**Dedicated Supp**	Not supported by any research networks, programs, or professional groups.		
**Endorsement**	Not recommended by clinical guidelines		
**Automation Flag**	Manually used		
**Tool Citations**	118	Reported in 1 study	
**Authors**	5	Sample Size = 175	
**Journal Impact**	5.35	Annals of emergency medicine	
**Phase of Evaluation**	**Level of Evidence**	**Grade**	**Evaluation Studies**
**Phase C: Before implementation Does the tool work? Is it possible?**	Internal validation	**C3**	Developed and internally validated: • Haydel & Shembekar, 2003 (47)
	External validation	**C2**	Not reported
	External validation multiple times	**C1**	Not reported
**Phase B: Planning for implementation: Is the tool practicable?**	Usability	**B3**	Not reported
	Potential effect	**B2**	Not reported
	Potential effect & Usability	**B1**	Not reported
**Phase A: After implementation: Is the tool desirable?**	Evaluation of post-implementation impact on Clinical Effectiveness, Patient Safety or Healthcare Efficiency	**A3**	No subjective studies are reported
		**A2**	No observational studies are reported
		**A1**	No experimental studies are reported
**Assigned Grade**	**Grade C3**		A1 A2 A3 B1 B2 B3 C1 C2 ●
**Justification**	Haydel rule was developed and tested successfully for internal validity in 2003 (47). The rule was not tested for external validity. It was not evaluated for usability, potential effect or post-implementation impact. Accordingly, the final grade assigned to Greenes rule is C3.		
**References**	Development and Internal Validation: • Haydel, M. J., & Shembekar, A. D. (2003). Prediction of intracranial injury in children aged five years and older with loss of consciousness after minor head injury due to nontrivial mechanisms. Annals of emergency medicine, 42(4), 507-514. Additional Commentary and Reviews:		

	• Maguire, J. L., Boutis, K., Uleryk, E. M., Laupacis, A., & Parkin, P. C. (2009). Should a head-injured child receive a head CT scan? A systematic review of clinical prediction rules. Pediatrics, 124(1), e145-e154.<br>• Maguire, J. L., Kulik, D. M., Laupacis, A., Kuppermann, N., Uleryk, E. M., & Parkin, P. C. (2011). Clinical prediction rules for children: a systematic review. Pediatrics, 128(3), e666-e677.<br>• Pickering, A., Harnan, S., Fitzgerald, P., Pandor, A., & Goodacre, S. (2011). Clinical decision rules for children with minor head injury: a systematic review. Archives of disease in childhood, 96(5), 414-421.<br>• Pandor, A., Goodacre, S., Harnan, S., Holmes, M., Pickering, A., Fitzgerald, P., ... & Stevenson, M. (2011). Diagnostic management strategies for adults and children with minor head injury: a systematic review and an economic evaluation. Health technology assessment (Winchester, England), 15(27), 1.<br>• Pandor, A., Harnan, S., Goodacre, S., Pickering, A., Fitzgerald, P., & Rees, A. (2012). Diagnostic accuracy of clinical characteristics for identifying CT abnormality after minor brain injury: a systematic review and meta-analysis. Journal of neurotrauma, 29(5), 707-718.
**Colour Code**	• Important Findings<br>• Less Relevant Findings<br>• Positive Findings<br>• Negative Findings

8.10. Atabaki Rule – Grade C3

Table 10: The GRASP Framework Detailed Report of Atabaki Rule

**Name**	Atabaki Rule for Paediatric Head Injury/Trauma		
**Authors/Year**	Dr. Shireen M. Atabaki, United States, 2008		
**Category**	Diagnostic		
**Intended use**	Identifies children at low risk for brain injuries after mild head trauma		
**Intended user**	Physicians		
**Clinical area**	Emergency department (ED)		
**Target Population**	Children less than 21 years of age at ED for head trauma		
**Target Outcome**	Traumatic brain injury		
**Action**	Do/Do Not Consider CT + Acute intervention		
**Input source**	Objective data (clinical examination) + subjective data (reported by child/parents)		
**Input type**	Clinical data: Mechanism of injury, loss of consciousness, amnesia, mental status change, lethargy, seizure, headache, vomiting, dizziness, drug or alcohol, sensory deficit, skull defect, basal skull fracture, scalp hematoma/laceration, and Glasgow coma scale score		
**Local context**	Input does not depend on local context of data		
**Methodology**	Recursive partitioning		
**Int. Validation**	Cross validation		
**Dedicated Supp**	Not supported by any research networks, programs, or professional groups.		
**Endorsement**	Not recommended by clinical guidelines		
**Automation Flag**	Manually used		
**Tool Citations**	111	Reported in 1 study	
**Authors**	8	Sample Size = 1,000	
**Journal Impact**	5.73	Archives of paediatrics & adolescent medicine	
**Phase of Evaluation**	**Level of Evidence**	**Grade**	**Evaluation Studies**
**Phase C: Before implementation**	Internal validation	**C3**	Developed and internally validated:<br>• Atabaki et al, 2008 (39)
	External validation	**C2**	Not reported

**Does the tool work? Is it possible?**	External validation multiple times	**C1**	Not reported
**Phase B:<br>Planning for implementation:<br>Is the tool practicable?**	Usability	**B3**	Not reported
	Potential effect	**B2**	Not reported
	Potential effect & Usability	**B1**	Not reported
**Phase A:<br>After implementation:<br>Is the tool desirable?**	Evaluation of post-implementation impact on Clinical Effectiveness, Patient Safety or Healthcare Efficiency	**A3**	No subjective studies are reported
		**A2**	No observational studies are reported
		**A1**	No experimental studies are reported
**Assigned Grade**	**Grade C3**		A1 | A2 | A3 | B1 | B2 | B3 | C1 | C2 | ●
**Justification**	Atabaki rule was developed and tested successfully for internal validity in 2008 (39). The rule was not tested for external validity. It was not evaluated for usability, potential effect or post-implementation impact. Accordingly, the final grade assigned to Atabaki rule is C3.		
**References**	Development and Internal Validation:<br>• Atabaki, S. M., Stiell, I. G., Bazarian, J. J., Sadow, K. E., Vu, T. T., Camarca, M. A., ... & Chamberlain, J. M. (2008). A clinical decision rule for cranial computed tomography in minor pediatric head trauma. Archives of pediatrics & adolescent medicine, 162(5), 439-445.<br>Systematic review studies:<br>• Maguire, J. L., Boutis, K., Uleryk, E. M., Laupacis, A., & Parkin, P. C. (2009). Should a head-injured child receive a head CT scan? A systematic review of clinical prediction rules. Pediatrics, 124(1), e145-e154.<br>• Maguire, J. L., Kulik, D. M., Laupacis, A., Kuppermann, N., Uleryk, E. M., & Parkin, P. C. (2011). Clinical prediction rules for children: a systematic review. Pediatrics, 128(3), e666-e677.<br>• Pickering, A., Harnan, S., Fitzgerald, P., Pandor, A., & Goodacre, S. (2011). Clinical decision rules for children with minor head injury: a systematic review. Archives of disease in childhood, 96(5), 414-421.<br>• Pandor, A., Goodacre, S., Harnan, S., Holmes, M., Pickering, A., Fitzgerald, P., ... & Stevenson, M. (2011). Diagnostic management strategies for adults and children with minor head injury: a systematic review and an economic evaluation. Health technology assessment (Winchester, England), 15(27), 1.<br>• Pandor, A., Harnan, S., Goodacre, S., Pickering, A., Fitzgerald, P., & Rees, A. (2012). Diagnostic accuracy of clinical characteristics for identifying CT abnormality after minor brain injury: a systematic review and meta-analysis. Journal of neurotrauma, 29(5), 707-718.<br>• Sempértegui Cárdenas, P. X. (2016). Validación de una escala de predicción de lesiones intracraneales para trauma cráneo-encefálico en niños de 0 a 5 años del Hospital Vicente Corral Moscoso Enero-Diciembre 2014. Estudio de test diagnóstico (Master's thesis).		
**Colour Code**	• Important Findings<br>• Less Relevant Findings	• Positive Findings<br>• Negative Findings	

8.11. Buchanich Rule – Grade C3

Table 11: The GRASP Framework Detailed Report of Buchanich Rule

**Name**	Buchanich Rule for Paediatric Head Injury/Trauma											
**Authors/Year**	Dr. Jeanine M. Buchanich, United States, 2007											
**Category**	Diagnostic											
**Intended use**	Identifies children at low risk for brain injuries after mild head trauma											
**Intended user**	Physicians											
**Clinical area**	Emergency department (ED)											
**Target Population**	Children less than three years of age at ED for head trauma											
**Target Outcome**	Traumatic brain injury											
**Action**	Do/Do Not Consider CT + Acute intervention											
**Input source**	Objective data (clinical examination) + subjective data (reported by child/parents)											
**Input type**	Clinical data: vision changes, scalp lacerations, history of vomiting, abnormal mental status, clinical signs of skull fracture, and headache.											
**Local context**	Input does not depend on local context of data											
**Methodology**	Recursive partitioning											
**Int. Validation**	Cross validation											
**Dedicated Supp**	Not supported by any research networks, programs, or professional groups.											
**Endorsement**	Not recommended by clinical guidelines											
**Automation Flag**	Manually used											
**Tool Citations**	4	Reported in 1 study										
**Authors**	1	Sample Size = 97										
**Journal Impact**	1	Doctoral dissertation, University of Pittsburgh										
**Phase of Evaluation**	**Level of Evidence**	**Grade**	**Evaluation Studies**									
**Phase C: Before implementation Does the tool work? Is it possible?**	Internal validation	**C3**	Developed and internally validated: • Buchanich, 2007 (40)									
	External validation	**C2**	Not reported									
	External validation multiple times	**C1**	Not reported									
**Phase B: Planning for implementation: Is the tool practicable?**	Usability	**B3**	Not reported									
	Potential effect	**B2**	Not reported									
	Potential effect & Usability	**B1**	Not reported									
**Phase A: After implementation: Is the tool desirable?**	Evaluation of post-implementation impact on Clinical Effectiveness, Patient Safety or Healthcare Efficiency	**A3**	No subjective studies are reported									
		**A2**	No observational studies are reported									
		**A1**	No experimental studies are reported									
**Assigned Grade**	**Grade C3**		A1	A2	A3	B1	B2	B3	C1	C2	●	
**Justification**	Buchanich rule was developed and tested successfully for internal validity in 2007 (40). The rule was not tested for external validity. It was not evaluated for usability, potential effect or post-implementation impact. Accordingly, the final grade assigned to Buchanich rule is C3.											
**References**	Development and Internal Validation: • Buchanich, J. M. (2007). A clinical decision-making rule for mild head injury in children less than three years old (Doctoral dissertation, University of Pittsburgh). Systematic review studies:											

	• Pickering, A., Harnan, S., Fitzgerald, P., Pandor, A., & Goodacre, S. (2011). Clinical decision rules for children with minor head injury: a systematic review. Archives of disease in childhood, 96(5), 414-421.<br>• Tavarez, M. M., Atabaki, S. M., & Teach, S. J. (2012). Acute evaluation of pediatric patients with minor traumatic brain injury. Current opinion in pediatrics, 24(3), 307-313.<br>• Pandor, A., Harnan, S., Goodacre, S., Pickering, A., Fitzgerald, P., & Rees, A. (2012). Diagnostic accuracy of clinical characteristics for identifying CT abnormality after minor brain injury: a systematic review and meta-analysis. Journal of neurotrauma, 29(5), 707-718.<br>• Shiomi, N., Echigo, T., Hino, A., Hashimoto, N., & Yamaki, T. (2016). Criteria for CT and initial management of head injured infants: A review. Neurologia medico-chirurgica, 56(7), 442-448.
**Colour Code**	• Important Findings<br>• Less Relevant Findings<br>• Positive Findings<br>• Negative Findings

8.12. Da Dalt Rule – Grade C0

Table 12: The GRASP Framework Detailed Report of Da Dalt Rule

**Name**	Da Dalt Rule for Paediatric Head Injury/Trauma		
**Authors/Year**	Dr. Liviana Da Dalt, Italy, 2006		
**Category**	Diagnostic		
**Intended use**	Predict the need for computed tomography among children with head trauma		
**Intended user**	Physicians		
**Clinical area**	Emergency department (ED)		
**Target Population**	Children less than 16 years at ED for head trauma		
**Target Outcome**	Traumatic brain injury		
**Action**	Do/Do Not Consider CT + Acute intervention		
**Input source**	Objective data (clinical examination) + subjective data (reported by child/parents)		
**Input type**	Clinical data: Loss of consciousness, prolonged headache, vomiting, Impact seizure, drowsiness, amnesia, abnormal neurological examination, lower Glasgow Coma Scale, and clinical evidence of basal or non-frontal skull fracture.		
**Local context**	Input does not depend on local context of data		
**Methodology**	Multivariate logistic regression analysis		
**Int. Validation**	Not reported		
**Dedicated Supp**	Not supported by any research networks, programs, or professional groups.		
**Endorsement**	Not recommended by clinical guidelines		
**Automation Flag**	Manually used		
**Tool Citations**	85	Reported in 1 study	
**Authors**	8	Sample Size = 3,806	
**Journal Impact**	1.79	European journal of paediatrics	
**Phase of Evaluation**	**Level of Evidence**	**Grade**	**Evaluation Studies**
**Phase C: Before implementation Does the tool work? Is it possible?**	Internal validation	**C3**	Developed but not tested for internal validity:<br>• Da Dalt et al, 2006 (41)
	External validation	**C2**	Not reported
	External validation multiple times	**C1**	Not reported
**Phase B:**	Usability	**B3**	Not reported
	Potential effect	**B2**	Not reported

**Planning for implementation: Is the tool practicable?**	Potential effect & Usability	**B1**	Not reported
**Phase A: After implementation: Is the tool desirable?**	Evaluation of post-implementation impact on Clinical Effectiveness, Patient Safety or Healthcare Efficiency	**A3**	No subjective studies are reported
		**A2**	No observational studies are reported
		**A1**	No experimental studies are reported
**Assigned Grade**	**Grade C0**		A1 A2 A3 B1 B2 B3 C1 C2 ○
**Justification**	Da Dalt rule was developed in 2006 but was not tested for internal validity (41). The rule was not tested for external validity. It was not evaluated for usability, potential effect or post-implementation impact. Accordingly, the final grade assigned to Da Dalt rule is C0.		
**References**	Development and Internal Validation:<br>• Da Dalt, L., Marchi, A. G., Laudizi, L., Crichiutti, G., Messi, G., Pavanello, L., ... & Barbone, F. (2006). Predictors of intracranial injuries in children after blunt head trauma. European journal of pediatrics, 165(3), 142-148. (Not tested for internal validity).<br>Additional Commentary and Reviews:<br>• Maguire, J. L., Boutis, K., Uleryk, E. M., Laupacis, A., & Parkin, P. C. (2009). Should a head-injured child receive a head CT scan? A systematic review of clinical prediction rules. Pediatrics, 124(1), e145-e154.<br>• Maguire, J. L., Kulik, D. M., Laupacis, A., Kuppermann, N., Uleryk, E. M., & Parkin, P. C. (2011). Clinical prediction rules for children: a systematic review. Pediatrics, 128(3), e666-e677.<br>• Pickering, A., Harnan, S., Fitzgerald, P., Pandor, A., & Goodacre, S. (2011). Clinical decision rules for children with minor head injury: a systematic review. Archives of disease in childhood, 96(5), 414-421.<br>• Pandor, A., Goodacre, S., Harnan, S., Holmes, M., Pickering, A., Fitzgerald, P., ... & Stevenson, M. (2011). Diagnostic management strategies for adults and children with minor head injury: a systematic review and an economic evaluation. Health technology assessment (Winchester, England), 15(27), 1.		
**Colour Code**	• Important Findings<br>• Less Relevant Findings		• Positive Findings<br>• Negative Findings

8.13. Greenes Rule – Grade C0

Table 13: The GRASP Framework Detailed Report of Greenes Rule

**Name**	Greenes Rule for Paediatrics Head Injury/Trauma
**Authors/Year**	Dr. David S. Greenes, United States, 2001
**Category**	Diagnostic
**Intended use**	Identifies infants at low risk for brain injuries after head trauma
**Intended user**	Physicians
**Clinical area**	Emergency department (ED)
**Target Population**	Infants less than two years of age at ED for head trauma
**Target Outcome**	Traumatic brain injury
**Action**	Do/Do Not Consider CT + Acute intervention
**Input source**	Objective data (clinical examination) + subjective data (reported by parents)
**Input type**	Clinical data: Age in months, scalp haematoma size, haematoma location.
**Local context**	Input does not depend on local context of data
**Methodology**	Multivariate logistic regression analysis

<table>
<tr><td>Int. Validation</td><td colspan="12">Not reported</td></tr>
<tr><td>Dedicated Supp</td><td colspan="12">Not supported by any research networks, programs, or professional groups.</td></tr>
<tr><td>Endorsement</td><td colspan="12">Not recommended by clinical guidelines</td></tr>
<tr><td>Automation Flag</td><td colspan="12">Manually used</td></tr>
<tr><td>Tool Citations</td><td>237</td><td colspan="11">Reported in 2 studies</td></tr>
<tr><td>Authors</td><td>2</td><td colspan="11">Sample Size = 422</td></tr>
<tr><td>Journal Impact</td><td>5.7</td><td colspan="11">Paediatrics</td></tr>
<tr><td>Phase of Evaluation</td><td>Level of Evidence</td><td>Grade</td><td colspan="10">Evaluation Studies</td></tr>
<tr><td rowspan="3">Phase C:
Before implementation
Does the tool work? Is it possible?</td><td>Internal validation</td><td>C3</td><td colspan="10">Developed but not tested for internal validity:
• Greenes & Schutzman, 1999 (44)
• Greenes & Schutzman, 2001 (45)</td></tr>
<tr><td>External validation</td><td>C2</td><td colspan="10">Not reported</td></tr>
<tr><td>External validation multiple times</td><td>C1</td><td colspan="10">Not reported</td></tr>
<tr><td rowspan="3">Phase B:
Planning for implementation:
Is the tool practicable?</td><td>Usability</td><td>B3</td><td colspan="10">Not reported</td></tr>
<tr><td>Potential effect</td><td>B2</td><td colspan="10">Not reported</td></tr>
<tr><td>Potential effect & Usability</td><td>B1</td><td colspan="10">Not reported</td></tr>
<tr><td rowspan="3">Phase A:
After implementation:
Is the tool desirable?</td><td rowspan="3">Evaluation of post-implementation impact on Clinical Effectiveness, Patient Safety or Healthcare Efficiency</td><td>A3</td><td colspan="10">No subjective studies are reported</td></tr>
<tr><td>A2</td><td colspan="10">No observational studies are reported</td></tr>
<tr><td>A1</td><td colspan="10">No experimental studies are reported</td></tr>
<tr><td>Assigned Grade</td><td colspan="2">Grade C0</td><td>A1</td><td>A2</td><td>A3</td><td>B1</td><td>B2</td><td>B3</td><td>C1</td><td>C2</td><td>O</td><td></td></tr>
<tr><td>Justification</td><td colspan="12">Greenes rule was developed in 2001 but was not tested for internal validity (44, 45). The rule was not tested for external validity. It was not evaluated for usability, potential effect or post-implementation impact. Accordingly, the final grade assigned to Greenes rule is C0.</td></tr>
<tr><td>References</td><td colspan="12">Development and Internal Validation:

• Greenes, D. S., & Schutzman, S. A. (1999). Clinical indicators of intracranial injury in head-injured infants. Pediatrics, 104(4), 861-867. (Not tested for internal validity).

• Greenes, D. S., & Schutzman, S. A. (2001). Clinical significance of scalp abnormalities in asymptomatic head-injured infants. Pediatric emergency care, 17(2), 88-92. (Not tested for internal validity).

Systematic review studies:

• Maguire, J. L., Boutis, K., Uleryk, E. M., Laupacis, A., & Parkin, P. C. (2009). Should a head-injured child receive a head CT scan? A systematic review of clinical prediction rules. Pediatrics, 124(1), e145-e154.

• Maguire, J. L., Kulik, D. M., Laupacis, A., Kuppermann, N., Uleryk, E. M., & Parkin, P. C. (2011). Clinical prediction rules for children: a systematic review. Pediatrics, 128(3), e666-e677.

• Pickering, A., Harnan, S., Fitzgerald, P., Pandor, A., & Goodacre, S. (2011). Clinical decision rules for children with minor head injury: a systematic review. Archives of disease in childhood, 96(5), 414-421.

• Pandor, A., Goodacre, S., Harnan, S., Holmes, M., Pickering, A., Fitzgerald, P., ... & Stevenson, M. (2011). Diagnostic management strategies for adults and children with minor head injury: a systematic review and an economic evaluation. Health technology assessment (Winchester, England), 15(27), 1.

• Sempértegui Cárdenas, P. X. (2016). Validación de una escala de predicción de lesiones intracraneales para trauma cráneo-encefálico en niños de 0 a 5 años del Hospital Vicente Corral Moscoso Enero-Diciembre 2014. Estudio de test diagnóstico (Master's thesis).</td></tr>
</table>

	• Bressan, S., Marchetto, L., Lyons, T. W., Monuteaux, M. C., Freedman, S. B., Da Dalt, L., & Nigrovic, L. E. (2017). A Systematic Review and Meta-Analysis of the Management and Outcomes of Isolated Skull Fractures in Children. Annals of emergency medicine.	
**Colour Code**	• Important Findings<br>• Less Relevant Findings	• Positive Findings<br>• Negative Findings

8.14. Klemetti Rule – Grade C0

Table 14: The GRASP Framework Detailed Report of Klemetti Rule

**Name**	Klemetti Rule for Paediatrics Head Injury/Trauma		
**Authors/Year**	Dr. Sanna Klemetti, Finland, 2009		
**Category**	Diagnostic		
**Intended use**	Identifies children at low risk for traumatic brain injuries after head trauma		
**Intended user**	Physicians		
**Clinical area**	Emergency department (ED)		
**Target Population**	Children less than 16 years of age at ED for head trauma		
**Target Outcome**	Traumatic brain injury		
**Action**	Do/Do Not Consider CT + Acute intervention		
**Input source**	Objective data (clinical examination) + subjective data (reported by child/parents)		
**Input type**	Clinical data: Abnormal mental status, signs of skull fracture, neurologic deficit, scalp trauma, loss of consciousness, and vertigo.		
**Local context**	Input does not depend on local context of data		
**Methodology**	Multivariate logistic regression analysis		
**Int. Validation**	Not reported		
**Dedicated Supp**	Not supported by any research networks, programs, or professional groups.		
**Endorsement**	Not recommended by clinical guidelines		
**Automation Flag**	Manually used		
**Tool Citations**	18	Reported in 1 study	
**Authors**	4	Sample Size = 485	
**Journal Impact**	1.07	Paediatric emergency care	
**Phase of Evaluation**	**Level of Evidence**	**Grade**	**Evaluation Studies**
**Phase C: Before implementation Does the tool work? Is it possible?**	Internal validation	**C3**	Developed but not tested for internal validity:<br>• Klemetti et al, 2009 (48)
	External validation	**C2**	Not reported
	External validation multiple times	**C1**	Not reported
**Phase B: Planning for implementation: Is the tool practicable?**	Usability	**B3**	Not reported
	Potential effect	**B2**	Not reported
	Potential effect & Usability	**B1**	Not reported
**Phase A: After implementation: Is the tool desirable?**	Evaluation of post-implementation impact on Clinical Effectiveness, Patient Safety or Healthcare Efficiency	**A3**	No subjective studies are reported
		**A2**	No observational studies are reported
		**A1**	No experimental studies are reported
**Assigned Grade**	**Grade C0**		A1 A2 A3 B1 B2 B3 C1 C2 O

**Justification**	Klemetti rule was developed in 2009 but was not tested for internal validity (48). The rule was not tested for external validity. It was not evaluated for usability, potential effect or post-implementation impact. Accordingly, the final grade assigned to Klemetti rule is C0.	
**References**	Development and Internal Validation:<br>• Klemetti, S., Uhari, M., Pokka, T., & Rantala, H. (2009). Evaluation of decision rules for identifying serious consequences of traumatic head injuries in pediatric patients. Pediatric emergency care, 25(12), 811-815. (Not tested for internal validity).<br>Additional Commentary and Reviews:<br>• Pickering, A., Harnan, S., Fitzgerald, P., Pandor, A., & Goodacre, S. (2011). Clinical decision rules for children with minor head injury: a systematic review. Archives of disease in childhood, 96(5), 414-421.<br>• Pandor, A., Goodacre, S., Harnan, S., Holmes, M., Pickering, A., Fitzgerald, P., ... & Stevenson, M. (2011). Diagnostic management strategies for adults and children with minor head injury: a systematic review and an economic evaluation. Health technology assessment (Winchester, England), 15(27), 1.<br>• Sempértegui Cárdenas, P. X. (2016). Validación de una escala de predicción de lesiones intracraneales para trauma cráneo-encefálico en niños de 0 a 5 años del Hospital Vicente Corral Moscoso Enero-Diciembre 2014. Estudio de test diagnóstico (Master's thesis).	
**Colour Code**	• Important Findings<br>• Less Relevant Findings	• Positive Findings<br>• Negative Findings

8.15. Quayle Rule – Grade C0

Table 15: The GRASP Framework Detailed Report of Quayle Rule

**Name**	Quayle Rule for Paediatrics Head Injury/Trauma		
**Authors/Year**	Dr. Kimberly S. Quayle, Unites States, 1997		
**Category**	Diagnostic		
**Intended use**	Identifies children at low risk for brain injuries after head trauma		
**Intended user**	Physicians		
**Clinical area**	Emergency department (ED)		
**Target Population**	Children less than 18 years of age at ED for head trauma		
**Target Outcome**	Traumatic brain injury		
**Action**	Do/Do Not Consider CT + Acute intervention		
**Input source**	Objective data (clinical examination) + subjective data (reported by child/parents)		
**Input type**	Clinical data: Altered mental status, focal neurologic deficit, seizure, signs of a basilar skull fracture, loss of consciousness for more than 5 minutes, and skull fracture.		
**Local context**	Input does not depend on local context of data		
**Methodology**	Multivariate logistic regression analysis		
**Int. Validation**	Not reported		
**Dedicated Supp**	Not supported by any research networks, programs, or professional groups.		
**Endorsement**	Not recommended by clinical guidelines		
**Automation Flag**	Manually used		
**Tool Citations**	291	Reported in 1 study	
**Authors**	7	Sample Size = 322	
**Journal Impact**	5.7	Paediatrics	
**Phase of Evaluation**	**Level of Evidence**	**Grade**	**Evaluation Studies**
**Phase C:**<br>**Before implementation**	Internal validation	**C3**	Developed but not tested for internal validity:<br>• Quayle et al, 1997 (53)

**Does the tool work? Is it possible?**	External validation	**C2**	Not reported
	External validation multiple times	**C1**	Not reported
**Phase B: Planning for implementation: Is the tool practicable?**	Usability	**B3**	Not reported
	Potential effect	**B2**	Not reported
	Potential effect & Usability	**B1**	Not reported
**Phase A: After implementation: Is the tool desirable?**	Evaluation of post-implementation impact on Clinical Effectiveness, Patient Safety or Healthcare Efficiency	**A3**	No subjective studies are reported
		**A2**	No observational studies are reported
		**A1**	No experimental studies are reported
**Assigned Grade**	**Grade C0**		A1 A2 A3 B1 B2 B3 C1 C2 ○
**Justification**	Dr. Kimberly Quayle in 1997 tried to develop a clinical prediction rule, to identify children at low risk for traumatic brain injuries after head trauma, through determining clinical signs and symptoms that can reliably predict an abnormality on cranial computed tomography (CT) (53). The study could not produce a predictive rule with sufficient internal validity. Accordingly, the final grade assigned to this rule is C0.		
**References**	Development and Internal Validation:<br>• Quayle, K. S., Jaffe, D. M., Kuppermann, N., Kaufman, B. A., Lee, B. C., Park, T. S., & McAlister, W. H. (1997). Diagnostic testing for acute head injury in children: when are head computed tomography and skull radiographs indicated?. Pediatrics, 99(5), e11-e11. (Not tested for internal validity).<br>Additional Commentary and Reviews:<br>• Pickering, A., Harnan, S., Fitzgerald, P., Pandor, A., & Goodacre, S. (2011). Clinical decision rules for children with minor head injury: a systematic review. Archives of disease in childhood, 96(5), 414-421.<br>• Pandor, A., Goodacre, S., Harnan, S., Holmes, M., Pickering, A., Fitzgerald, P., ... & Stevenson, M. (2011). Diagnostic management strategies for adults and children with minor head injury: a systematic review and an economic evaluation. Health technology assessment (Winchester, England), 15(27), 1.		
**Colour Code**	• Important Findings<br>• Less Relevant Findings	• Positive Findings<br>• Negative Findings	

8.16. Dietrich Rule – Grade C0

Table 16: The GRASP Framework Detailed Report of Dietrich Rule

**Name**	Dietrich Rule for Paediatrics Head Injury/Trauma
**Authors/Year**	Dr. Ann Dietrich, United States, 1993
**Category**	Diagnostic
**Intended use**	Identifies children at low risk for brain injuries after head trauma
**Intended user**	Physicians
**Clinical area**	Emergency department (ED)
**Target Population**	Children less than 21 years of age at ED for head trauma
**Target Outcome**	Traumatic brain injury
**Action**	Do/Do Not Consider CT + Acute intervention
**Input source**	Objective data (clinical examination) + subjective data (reported by child/parents)
**Input type**	Clinical data: e.g. Loss of consciousness, clinical signs of focal neuro-deficits, seizures, and history of vomiting and headache.
**Local context**	Input does not depend on local context of data

**Methodology**	Multivariate logistic regression analysis		
**Int. Validation**	Not reported		
**Dedicated Supp**	Not supported by any research networks, programs, or professional groups.		
**Endorsement**	Not recommended by clinical guidelines		
**Automation Flag**	Manually used		
**Tool Citations**	220	Reported in 1 study	
**Authors**	5	Sample Size = 324	
**Journal Impact**	5.35	Annals of emergency medicine	
**Phase of Evaluation**	**Level of Evidence**	**Grade**	**Evaluation Studies**
**Phase C: Before implementation Does the tool work? Is it possible?**	Internal validation	**C3**	Developed but not tested for internal validity:<br>• Dietrich et al, 1993 (42)
	External validation	**C2**	Not reported
	External validation multiple times	**C1**	Not reported
**Phase B: Planning for implementation: Is the tool practicable?**	Usability	**B3**	Not reported
	Potential effect	**B2**	Not reported
	Potential effect & Usability	**B1**	Not reported
**Phase A: After implementation: Is the tool desirable?**	Evaluation of post-implementation impact on Clinical Effectiveness, Patient Safety or Healthcare Efficiency	**A3**	No subjective studies are reported
		**A2**	No observational studies are reported
		**A1**	No experimental studies are reported
**Assigned Grade**	**Grade C0**	A1 A2 A3 B1 B2 B3 C1 C2 O	
**Justification**	Dr. Ann Dietrich in 1993 tried to develop a clinical prediction rule, to identify children at low risk for traumatic brain injuries after head trauma, through determining clinical factors that reliably predict an abnormality on computed tomography (CT) (42). Dr. Dietrich study could not demonstrate a good correlation between the clinical symptoms of significant traumatic brain injury and the findings on the CT. The proposed rule did not have sufficient internal validity to be tested for external validity or to be implemented. Accordingly, the final grade assigned to this rule is C0.		
**References**	Development and Internal Validation:<br>• Dietrich, A. M., Bowman, M. J., Ginn-Pease, M. E., Kosnik, E., & King, D. R. (1993). Pediatric head injuries: can clinical factors reliably predict an abnormality on computed tomography?. Annals of emergency medicine, 22(10), 1535-1540. (Not tested for internal validity).<br><br>Additional Commentary and Reviews:<br>• Pickering, A., Harnan, S., Fitzgerald, P., Pandor, A., & Goodacre, S. (2011). Clinical decision rules for children with minor head injury: a systematic review. Archives of disease in childhood, 96(5), 414-421.<br>• Pandor, A., Goodacre, S., Harnan, S., Holmes, M., Pickering, A., Fitzgerald, P., ... & Stevenson, M. (2011). Diagnostic management strategies for adults and children with minor head injury: a systematic review and an economic evaluation. Health technology assessment (Winchester, England), 15(27), 1.		
**Colour Code**	• Important Findings<br>• Less Relevant Findings	• Positive Findings<br>• Negative Findings	

8.17. Güzel Rule – Grade C0

Table 17: The GRASP Framework Detailed Report of Güzel Rule

**Name**	Güzel Rule for Paediatrics Head Injury/Trauma		
**Authors/Year**	Dr. Ahmet Güzel, Turkey, 2009		
**Category**	Diagnostic		
**Intended use**	Identifies children at low risk for traumatic brain injuries after head trauma		
**Intended user**	Physicians		
**Clinical area**	Emergency department (ED)		
**Target Population**	Children less than 15 years of age at ED for head trauma		
**Target Outcome**	Traumatic brain injury		
**Action**	Do/Do Not Consider CT + Acute intervention		
**Input source**	Objective data (clinical examination) + subjective data (reported by child/parents)		
**Input type**	Clinical data: cause of injury, headache, post-traumatic amnesia, loss of consciousness, blurred vision, seizures, head lacerations, scalp haematoma, periorbital ecchymosis, otorrhea, skull fractures, and abnormal neurological findings.		
**Local context**	Input does not depend on local context of data		
**Methodology**	Multivariate logistic regression analysis		
**Int. Validation**	Not reported		
**Dedicated Supp**	Not supported by any research networks, programs, or professional groups.		
**Endorsement**	Not recommended by clinical guidelines		
**Automation Flag**	Manually used		
**Tool Citations**	17	Reported in 1 study	
**Authors**	6	Sample Size = 916	
**Journal Impact**	1	Paediatric neurosurgery	
**Phase of Evaluation**	**Level of Evidence**	**Grade**	**Evaluation Studies**
**Phase C: Before implementation Does the tool work? Is it possible?**	Internal validation	**C3**	Developed but not tested for internal validity: • Güzel et al, 2009 (46)
	External validation	**C2**	Not reported
	External validation multiple times	**C1**	Not reported
**Phase B: Planning for implementation: Is the tool practicable?**	Usability	**B3**	Not reported
	Potential effect	**B2**	Not reported
	Potential effect & Usability	**B1**	Not reported
**Phase A: After implementation: Is the tool desirable?**	Evaluation of post-implementation impact on Clinical Effectiveness, Patient Safety or Healthcare Efficiency	**A3**	No subjective studies are reported
		**A2**	No observational studies are reported
		**A1**	No experimental studies are reported
**Assigned Grade**	**Grade C0**		A1 A2 A3 B1 B2 B3 C1 C2 ○
**Justification**	Dr. Ahmet Güzel in 2009 tried to develop a clinical prediction rule, to identify children at low risk for traumatic brain injuries after head trauma, through determining clinical risk factors that can be used as predictors of abnormalities in cranial computed tomography scans following minor head injury. The study could not produce a predictive rule with sufficient internal validity (46). Accordingly, the final grade assigned to this rule is C0.		
**References**	Development and Internal Validation: • Güzel, A., Hiçdönmez, T., Temizöz, O., Aksu, B., Aylanç, H., & Karasalihoglu, S. (2009). Indications for brain computed tomography and hospital admission in		

	pediatric patients with minor head injury: how much can we rely upon clinical findings?. Pediatric neurosurgery, 45(4), 262-270. (Not tested for internal validity).<br><br>Additional Commentary and Reviews:<br><br>• Pickering, A., Harnan, S., Fitzgerald, P., Pandor, A., & Goodacre, S. (2011). Clinical decision rules for children with minor head injury: a systematic review. Archives of disease in childhood, 96(5), 414-421.<br><br>• Pandor, A., Goodacre, S., Harnan, S., Holmes, M., Pickering, A., Fitzgerald, P., ... & Stevenson, M. (2011). Diagnostic management strategies for adults and children with minor head injury: a systematic review and an economic evaluation. Health technology assessment (Winchester, England), 15(27), 1.	
**Colour Code**	• Important Findings<br>• Less Relevant Findings	• Positive Findings<br>• Negative Findings
